# Growth strategy of aerial green algae on building materials in the temperate climate zone and its relevance to substrate biodeterioration

**DOI:** 10.1038/s41598-025-31926-x

**Published:** 2025-12-10

**Authors:** Paulina Nowicka-Krawczyk, Michał Komar, Beata Gutarowska, Joanna Żelazna-Wieczorek, Izabela A. Jesionowska, Sława Glińska, Łucja Balcerzak

**Affiliations:** 1 Department of Algology and Mycology, Faculty of Biology and Environmental Protection, University of Lodz, Banacha 12/16, Lodz, 90- 237 Poland; 2 Department of Environmental Biotechnology, Faculty of Biotechnology and Food Sciences, Lodz University of Technology, Wólczańska 171/173, Lodz, 90-530 Poland; 3 Interdisciplinary Doctoral School, Lodz University of Technology, Żeromskiego 116, Lodz, 90-924 Poland; 4 Laboratory of Microscopic Imaging and Specialized Biological Techniques, University of Lodz, Banacha 12/16, Lodz, 90-237 Poland

**Keywords:** Algal biofilm, Aerial algae, Biodeterioration, Building materials, Phototrophic colonisation, Ecology, Ecology, Environmental sciences, Microbiology

## Abstract

Aerial green algae colonising building materials in temperate climates form complex biofilms that contribute to the deterioration of man-made structures, reducing their aesthetic value. The present study seeks to address whether algae, already at an early stage of colonisation, can adversely influence building substrates, contributing to biodeterioration beyond mere aesthetic impact. Early-stage colonisers – *Chloroidium saccharophilum*, *Klebsormidium nitens*, *Bracteacoccus minor*, *Diplosphaera chodatii*, and *Stichococcus bacillaris*, were isolated from brick and plaster walls, identified using traditional and molecular methods, and grown on experimental brick and plaster substrates. Over six months, algal growth in laboratory and environmental conditions was visualised and monitored by measuring chlorophyll a concentration, fluorescence intensity, and substrate profile height. All taxa were able to grow on both types of substrates at their surface and in the interior part. Most of them formed compact biofilms that filled substrate pores and overgrew the cavities, with some producing membrane-like layers (*K. nitens* and *S. bacillaris*). Algae with thick cell walls (*C. saccharophilum*, *D. chodatii*, *S. bacillaris*) and filamentous *K. nitens* detached the mineral grains from the brick surface during biofilm separation, as a consequence of prolonged colonisation over time. Except for *B. minor*, all taxa contributed to the geophysical biodeterioration of brick.

**Supplementary Information:**

The online version contains supplementary material available at 10.1038/s41598-025-31926-x.

## Introduction

Phototrophic biofilms in the terrestrial environment of the temperate climate zone can grow on various substrates, including both natural and man-made materials, forming a green surface layer^1^. The fundamental component of the biofilm is aerial green microalgae^2^. These microorganisms have adapted to living in a terrestrial environment, and their growth depends on the presence of water vapour and nutrients, both from the surfaces they colonise and from dust/aerosols in the air^3^. Colonisation of new spots is somewhat accidental – cells are spread by wind from one place to another, and after 40–60 days, a ‘green biofilm’ is formed^4^. Mature biofilms are complex in terms of biodiversity. Next to the dominant photobiont, they are composed of bacteria, cyanobacteria, algae, and fungi embedded in an extracellular polymeric substance (EPS) matrix^5–7^, and they coexist to protect the biofilm against severe external conditions.

The role of bacteria and fungi in the process of material deterioration has formed the basis of many previous studies due to their heterotrophic nature and the ability to chemically decompose colonised substrates^8,9^. Mature biofilms impact the structure of mineral materials not only through biofilm formation but also through the secretion of inorganic/organic acids and salt crystallisation. Microbial-induced mobilisation of ions leads to superficial or internal deposits, weakening the structure and decreasing the technical state of materials^10,11^. Ceramic materials, like bricks, have a long history of use — more than 8000 years^12^. They serve as the basic construction material for many cultural relics and significant historical sites^11^; therefore, understanding microbial degradation mechanisms is essential to plan conservation practices that adequately protect these sites. Nowadays, walls are often plastered for economic and decorative reasons. The high porosity of plaster enhances microbial colonisation, and the main symptoms of changes in material properties due to biofilm formation are: an increase in sorptivity and thermal rate, and a decrease in the diffusion of water vapor. Over time, microbial colonization strains the plaster structure, leading to microcracks and reduced plaster durability^13^.

Although some authors neglect the role of aerial algae in biodeterioration and believe their negative impact is purely aesthetic^14,15^, there are some reports on the geochemical deterioration potential of terrestrial green algae^16^. In theory, the life strategy of aerial algal cells suggests that their contribution to biodeterioration processes may be as direct as that of bacteria and fungi^3^. Therefore, there is reason to believe that algae are directly involved in the deterioration of materials to a greater extent than through aesthetic effects alone. The question arises, when do the first symptoms of algal-based direct deterioration occur? Can particular aerial green algal taxa, through early-stage biofilm formation, contribute to the deterioration of the structural integrity of the material?

To address this hypothesis and questions, algae from early-stage biofilms on brick and plaster – common construction and decorative facade materials in temperate climate – were collected. Through (1) the traditional and molecular identification of the ‘first’ colonisers of brick and plaster, (2) their cultivation on mineral substrates *in situ* and *ex situ* conditions for the biofilm formation, (3) the assessment of the algal growth rate, ability to penetrate the substrate and examination of substrates profile, and finally (4) visualisation of the biofilms impact on brick and plaster surface, the biodeterioration potential of studied species has been demonstrated.

## Results

### Aerial green algal colonisers in young biofilms

Algal biofilms in an early stage of colonisation, characterised by uniform structure and barely visible as greenish staining (‘young’ biofilms), were sampled from eight walls made of brick and twelve covered with plaster. Sampling sites varied in illumination (4.5%–86.1%), and substrates exhibited different moisture levels (0%–14%) (Supplementary Table S1). As a result of the field survey, 61 well-established strains of four morphotypes were obtained – 8 coccal with circular shape, 26 coccal and more/less oval, 22 coccal and more/less elongated, and 5 filamentous. Combined molecular analyses of nuclear and chloroplast markers, together with morphological and ultrastructural data, confirmed the presence of seven green algae: six from Chlorophyta and one from Charophyta. Charophyta was represented by the filamentous genus *Klebsormidium*, while Chlorophyta by: the spherical morphotype genus *Bracteacoccus*, oval-celled morphotype genera of *Chloroidium* and *Diplosphaera*, and elongated morphotypes of *Stichococcus*, *Pseudostichococcus*, and *Deuterostichococcus* genera. The origin of strains from biofilm samples, as well as the molecular distance matrices between strains, the morphometrics of strains, and details on the molecular records used in phylogenetic analyses are presented in Supplementary Tables S1–S4.

**Fig. 1:**
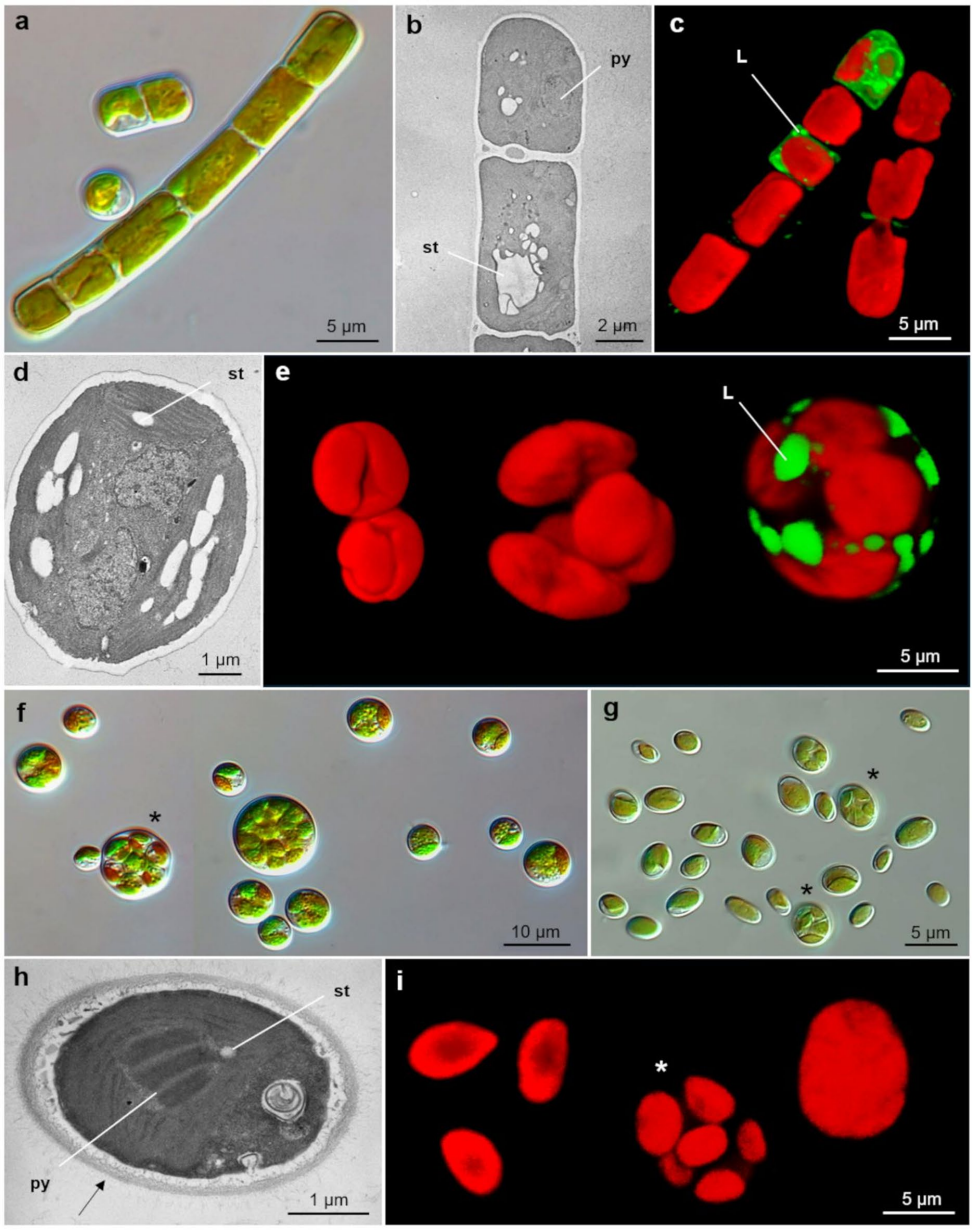
Microphotographs of green algae isolated from the biofilm samples, except the members from Prasiola group in light microscopy (LM), confocal laser-scanning microscopy (CLSM), and transmission electron microscopy (TEM): (**a–c**) *Klebsormidium nitens* PNK013 in (**a**) LM, (**b**) TEM, where (st) points at starch grains, while (py) at pyrenoids, and (**c**) CLSM, where (L) points at lipids; (**d–f**) *Bracteacoccus minor* PNK015 in (**d**) TEM, where (st) points at starch grains, (**e**) CLSM, where (L) points at lipids, and (**f**) LM, where (*) points at sporangia; (**g–i**) *Chloroidium saccharophilum* PNK010 in (**g**) LM, where (*) points at sporangia, (**h**) TEM, where (st) points at starch grains, while (py) at pyrenoids, and (**i**) CLSM, where (*) points at sporangia.

*Klebsormidium* (Klebsormidiales, Charophyta) strains form long, thin to medium filaments ((4.6) 5.5–6.2 (7.3) µm) with cells having a length/width ratio of (0.85) 1.09–1.48 (2.46) (Fig. 1a, b). Filaments easily break into short fragments or unicells (Fig. 1a). In young filaments, the chloroplast is lobed and fills most of the cell, whereas in older cells it reduces to about half and becomes less lobate with an uneven margin. Starch grains are near a small pyrenoid, and lipids accumulate as the cells mature (Fig. 1b, c). The isolated strains are molecularly highly similar (> 99.47%) and form a well-supported clade (BS ≥ 96 and PP = 1.00) with an epitype strain of *Klebsormidium nitens* SAG 13.92 and other strains of *K. nitens*, also strongly supported in the BI analysis (Supplementary Fig. S1).

*Bracteacoccus* (Sphaeropleales, Chlorophyta) strains form smooth, wavy, and convex-edged colonies of spherical cells with thin and smooth cell walls and variable diameters ((5.4) 6.2–9.9 (17.8) µm) (Fig. 1d, f). Reproduction occurs mainly *via* thick-walled autosporangia containing 3–6 autospores (Fig. 1f), though zoospores may occasionally form. The chloroplast is initially single and bilobed, later dividing into several plate-like forms without pyrenoids; starch grains and lipid droplets are often present (Fig. 1d, e). The isolated strains show high molecular similarity (> 99.66%) and cluster in a maximum-supported clade (BS = 100 and PP = 1.00) with the type strain of *Bracteacoccus minor* UTEX 66 and other strains of *B. minor* (Supplementary Fig. S2).

*Chloroidium* (Watanabeales, Chlorophyta) strains form smooth-edged colonies of oval cells, about twice as long as wide ((0.96) 1.56–1.82 (2.24); (4.6) 5.6–6.8 (9.0) × (2.5) 3.2–4.2 (5.2) µm) (Fig. 1g, h). Reproduction occurs *via* autosporangia producing 4–8 autospores (Fig. 1g, i). The single band-shaped and slightly concave chloroplast, occupying about half the cell volume, contains a large pyrenoid surrounded by starch grains in mature cells (Fig. 1h, i). The cell wall is thick, usually 200–250 nm wide (Fig. 1h). The isolated strains show high molecular similarity (> 99.48%) and form a maximally supported clade (BS = 100 and PP = 1.00) with *Chloroidium saccharophilum* strains, including the SAG 211-9a strain (Supplementary Fig. S3).

The most numerous were the members from the Prasiola group (Prasiolales, Chlorophyta). High morphological similarity of strains – all morphotypes were ‘*Stichocccus*-like’ – made the identification difficult; therefore, only the phylogeny allowed for delimiting the strains into species. The phylogeny tree is presented in Supplementary Figure S4.

**Fig. 2:**
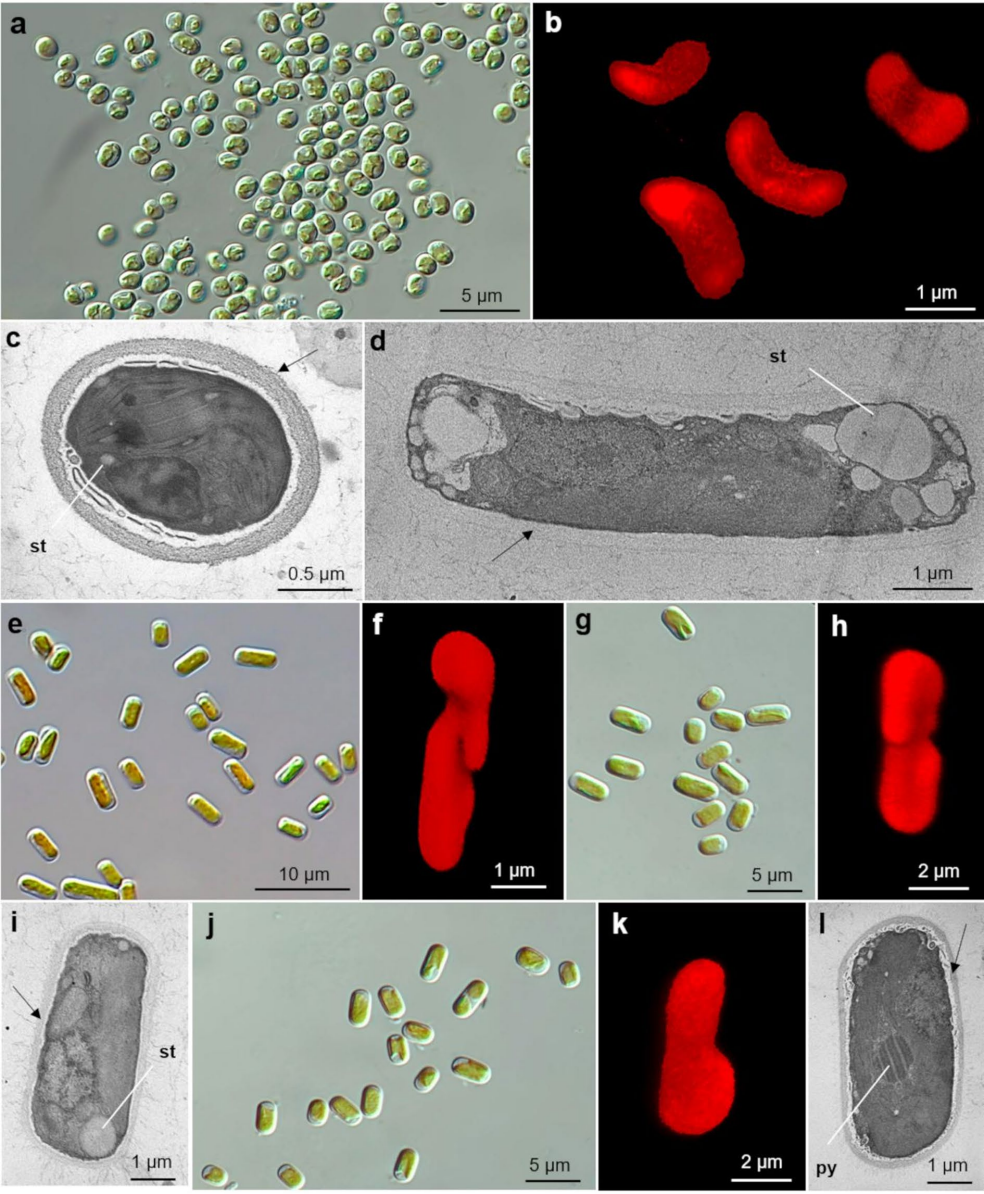
Microphotographs of green algae from the Prasiola group isolated from the biofilm samples in light microscopy (LM), confocal laser-scanning microscopy (CLSM), and transmission electron microscopy (TEM): (**a–c**) *Diplosphaera chodatii* PNK021 in (**a**) LM, (**b**) CLSM, and (**c**) TEM, where (st) points at starch grains, while an arrow at thick cell wall; (**d–f**) *Stichococcus bacillaris* PNK040 in (**d**) TEM where (st) points at starch grains, while an arrow at thick cell wall, (**e**) LM, and (**f**) CLSM; (**g–i**) *Pseudostichococcus monnalantoides* PNK007 in (**g**) LM, (**h**) CLSM, and (**i**) TEM, where (st) points at starch grains, while an arrow at thick cell wall; (**j–l**) *Deuterostichococcus epilithicus* PNK056 in (**j**) LM, (**k**) CLSM, and (**l**) TEM, where (st) points at starch grains, while an arrow at thick cell wall.

*Diplosphaera* strains form dense, shiny, and intense green colonies of small oval cells ((2.8) 3.3–4.1 (4.7) × (2.5) 2.8–3.1 (3.7) µm; length/width ratio (0.98) 1.17–1.37 (1.71)) (Fig. 2a, c). Each cell contains a single, slightly arched, and concave chloroplast – the LM view may give the impression of a double, parietal chloroplast at opposite sites (Fig. 2a, b) – with one pyrenoid and few starch grains; the cell wall is remarkably thick relative to cell size (Fig. 2c). The isolated strains exhibit high molecularly similarity (> 99.32%) and form a maximally supported clade (BS = 100 and PP = 1.00) with *Diplosphaera chodatii* strains.

*Stichococcus* strains form dense, dull-green colonies with a shiny surface. Cells are elongated ((3.8) 4.7–6.0 (10.0) × (2.0) 2.4–2.7 (3.2) µm) and up to four times longer than wide ((1.57) 1.81–2.40 (3.89)) (Fig. 2d, e). Each cell contains a single, slightly twisted chloroplast with irregular margins (Fig. 2f) and one pyrenoid; starch grains accumulate at the polar sites, and the cell wall is thick (Fig. 2d). The isolated strains show high molecular similarity (> 97.99%) and form a maximally supported clade (BS = 100 and PP = 1.00) with the epitype *Stichococcus bacillaris* SAG 335-8 and other strains of *S. bacillaris*.

*Pseudostichococcus* strains form dense, shiny green colonies of elongated cells ((4.9) 6.7–8.5 (11.3) × (2.7) 3.2–3.6 (4.3) µm) and up to three times longer than wide ((1.51) 1.90–2.46 (3.23)) (Fig. 2g, i). Cells possess a single, slightly twisted chloroplast (Fig. 2h) with one pyrenoid, large polar starch grains, and a thick cell wall (Fig. 2i). The isolated strains are molecularly highly similar (> 97.73%) and form a maximally supported clade (BS = 100 and PP = 1.00) with *Pseudostichococcus monallantoides* strains, showing an internal dichotomy: isolate PNK007 clusters with the epitype UTEX 2249 strain, while others group with SAG 380-1 strain.

*Deutrostichococcus* strains form dense, shiny green colonies of slightly elongated cells ((5.2) 6.5–8.3 (11.6) × (2.8) 3.2–3.4 (4.1) µm), and up to three times longer than wide ((1.62) 1.97–2.53 (3.25)) (Fig. 2j, l). Cells possess a single chloroplast, concave at approximately one-third of its length or in the middle (Fig. 2k, l), one pyrenoid, and starch grains in mature, long cells; the cell wall is thick (Fig. 2l). The isolated strains shows high molecular similarity (> 99.69%) and from a maximally supported clade (BS = 100 and PP = 1.00) with the epitype *Deuterostichococcus epilithicus* SAG 2060 strain and other strains of *D. epilithicus.*

Since the isolates of the Prasiola group resemble high morphological similarity and belong to the group of very low genetic variability^17^, only two taxa that are distinct in morphology, particularly at L/W ratio, and resemble the highest genetic distance were chosen for further experimentation on algal growth at brick and plaster substrates – *Diplosphaera chodatii* and *Stichococcus bacillaris*.

### Notes on algal occurrence among sampling sites

All of the identified algal species grow on the brick and plaster; however, *Diplosphaera chodatii* was predominantly found on bricks and was present only in one biofilm on plaster (Supplementary Table S1). The members of the Prasiola group were most frequent and absent at two sampling sites; however, this absence has no apparent explanation in terms of environmental traits (Supplementary Table S1).

**Fig. 3:**
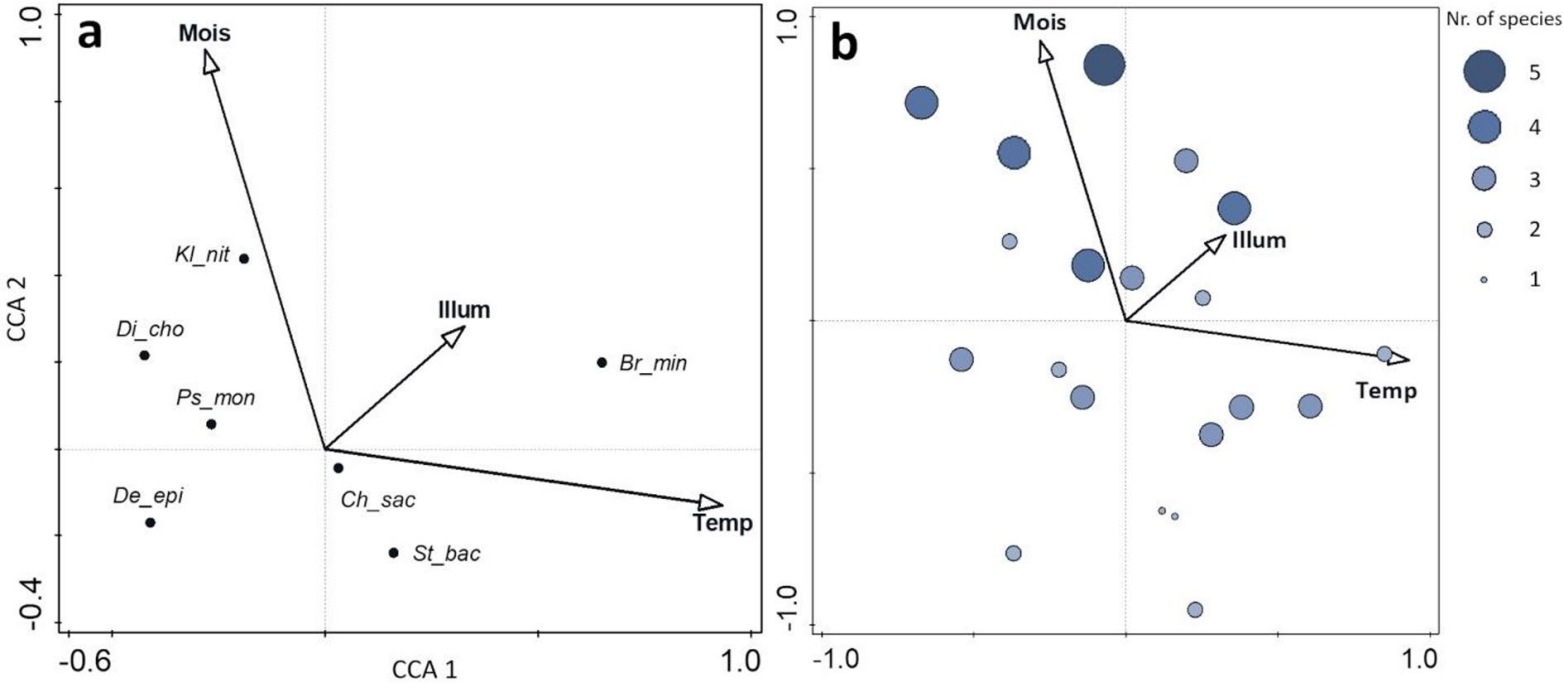
The Canonical Correspondence Analysis (CCA) ordination plots summarising the distribution of algal taxa across the most critical environmental variables for algal colonisation, (**a**) relations between individual taxa and factors, (**b**) diversity plot with the number of species at sampling sites; vectors of variables are: (Temp) substrate temperature, (Mois) substrate moisture, (Illum) percentage of illumination, and species: (Br_min) *Bracteacoccus minor*, (Ch_sach) *Chloroidium saccharophilum*, (De_epi) *Deuterostichococcus epilithicus*, (Di_cho) *Diplosphaera chodatii*, (Kl_nit) *Klebsormidium nitens*, (Ps_mon) *Pseudostichococcus monallantoides*, (St_bac) *Stichococcus bacillaris*; the analysis explained 90.73% of the total variation among the variables, and an Interactive Forward Selection Test selected the above variables with the contribution of: Temp – 48.7%, Mois – 23.1%, and Illum – 15.3%.

The Canonical Correspondence Analysis (CCA), used to trace the algal occurrence among environmental variables, reveals that the presence of *Bracteacoccus minor* is more closely correlated with elevated substrate temperature and illumination than with higher substrate moisture (Fig. 3a). In contrast, *Klebsormidium nitens* prefers more humid sites with moderate illumination. The *Chloroidium saccharophilum* occurrence is not affected by the changes in the analysed factors, as it is located closest to the beginning of the analysis’s ordination space. Among the Prasiola members, there is no clear ecological tendency; however, the CCA placed taxa selected previously for further experiment on biofilm growth at brick and plaster substrates (*Diplosphaera chodatii* and *Stichococcus bacillaris*) at extreme ends of the horizontal and vertical axes (Fig. 3a). Together with their morphological and molecular distinction, this ecological trait additionally supports the choice of these taxa among Prasiolales for further study.

There is no clear tendency or preference between species for co-occurrence in the biofilm, except that if the young biofilm is unialgal, then it is composed of *Chloroidium saccharophilum* (Supplementary Table S1). Furthermore, the CCA indicates that the moisture of the substrate and the percentage of illuminance increase algal diversity (Fig. 3b). Nevertheless, the moisture of the substrate contributed more to data variance (23.1%) than illumination (15.3%).

### The growth strategy of aerial algal colonisers

To assess algal growth rate on experimental brick (B) and plaster (P) in the laboratory (L) and environment (E), algal biomass was measured *via* chlorophyll a (chl-*a*) concentration (C_chla_).

**Fig. 4:**
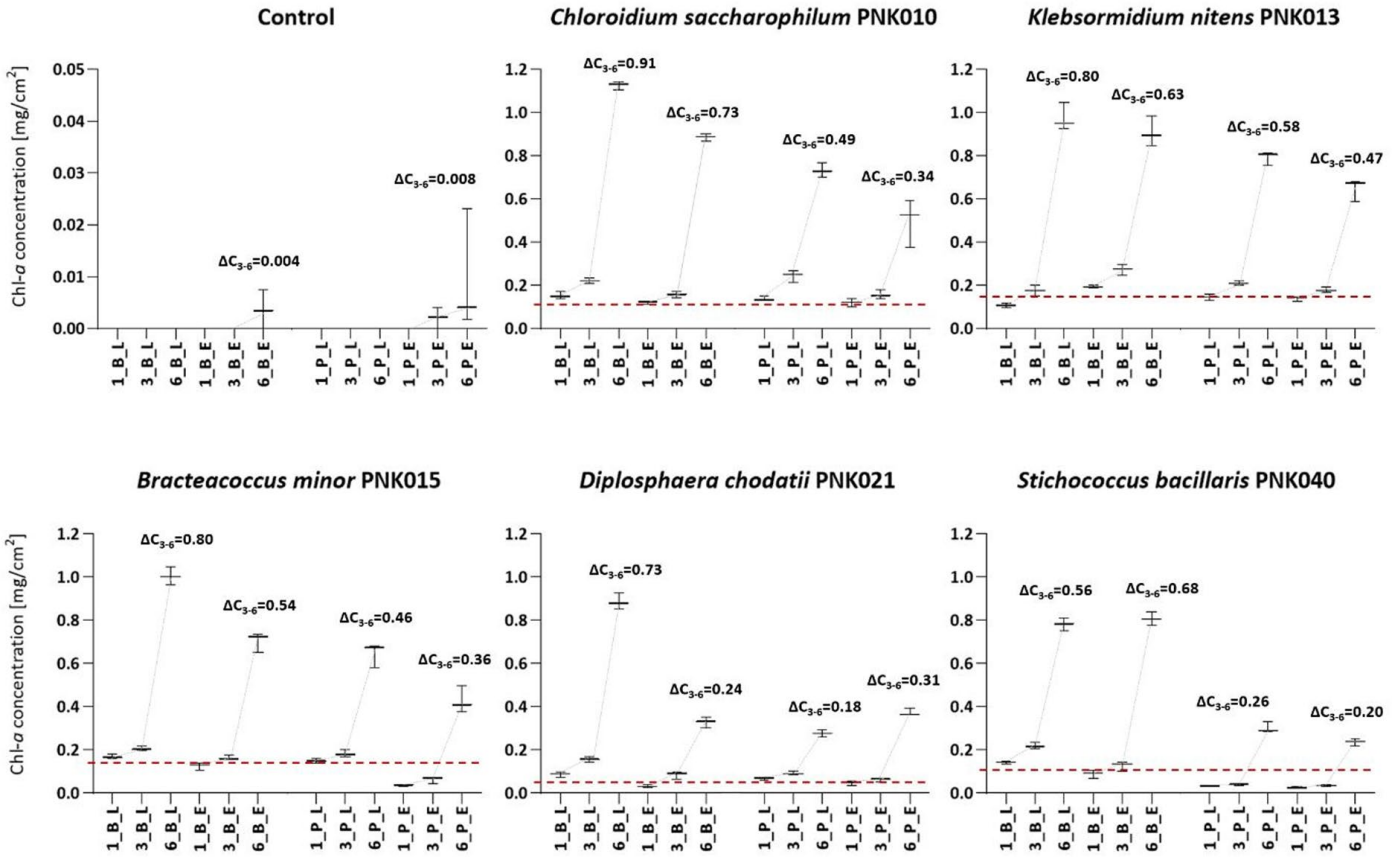
Changes in the chl-*a* concentration (C_chla_) at experimental brick (B) and plaster (P) after one (1), three (3) and six (6) months of cultivation in laboratory (L) and environmental (E) conditions, the dashed lines show the mean C_chla_ of the particular taxa inoculation culture (OD = 1.00) per 1 cm^2^ of the substrate and a delta of mean C_chla_ between the 3^rd^ and 6^th^ months (ΔC_3 − 6_) was added.

The control substrates remained uncontaminated during the experiment in the laboratory; however, in the environment, some amounts of chl-*a* were detected at the end of cultivation (C_chla_ B_E = 0.004 mg/cm^2^, C_chla_ P_E = 0.01 mg/cm^2^), but without any visual signs of algal colonisation (Fig. 4, Supplementary Fig. S5).

*C*. *saccharophilum* showed the greatest adaptation to the experimental substrates after inoculation. On both types of substrates, regardless of the cultivation location, the C_chla_ increased at substrates already after the first month in relation to the inoculum biomass (by: C_chla_ B_L = 0.04 mg/cm^2^, C_chla_ B_E = 0.006 mg/cm^2^, C_chla_ P_L = 0.02 mg/cm^2^, C_chla_ P_E = 0.003 mg/cm^2^) (Fig. 4). In *K. nitens*, in the first month, at both plaster substrates and the brick kept in the laboratory, a slight decrease in C_chla_ was noted (C_chla_ P_L/E = −0.03 mg/cm^2^, C_chla_ B_L = −0.06 mg/cm^2^) – only at the brick in situ, this alga at once adapted to growth (C_chla_ B_E = 0.02 mg/cm^2^). Other algae easily adapted to brick substrates in the laboratory; however, in the environment, a decrease in C_chla_ was observed, with the greatest decrease for *D. chodatii* (by 46% of the inoculum, C_chla_ B_E = −0.03 mg/cm^2^) (Fig. 4, Supplementary Tables S6–S7). Considering the plaster, in the laboratory, both *B. minor* and *D. chodatii* maintained their biomass at a similar level to the inoculum. However, in *S. bacillaris*, the C_chla_ gradually decreased by 69% of the inoculum biomass by the 3^rd^ month (C_chla_ P_L = −0.07 mg/cm^2^). The plaster kept in the environment was not easily overgrown by coccal algae. Except for *C. saccharophilum*, a decrease in C_chla_ was observed in all biofilms after one month. In *B. minor* and *S. bacillaris*, this decrease persisted until the 3^rd^ month, resulting in 57% (C_chla_ P_E = −0.09 mg/cm^2^ and 66% (C_chla_ P_E = −0.07 mg/cm^2^) reductions of the inoculum biomass, respectively (Fig. 4, Supplementary Tables S6–S7).

Both the visual appearance of the biofilms at experimental substrates (Supplementary Figs. S6–S10) and the C_chla_ measurements (Fig. 4, Supplementary Table S7) indicate that the biomass of algae on the brick substrate was higher than on the plaster. In general, samples cultivated in the laboratory were colonised to a greater extent than in the environment. The highest growth rate between the 3^rd^ and 6^th^ months, both *in situ *and *ex situ*, was observed at brick substrates with *C. saccharophilum*, while at plaster substrates, it was with *K. nitens*.

To examine the algal potential to penetrate substrates, the fluorescence intensity of chlorophyll pigments (^chl^FI) was measured at the cross-sections of the materials using confocal laser-scanning microscopy (CLSM).

**Fig. 5:**
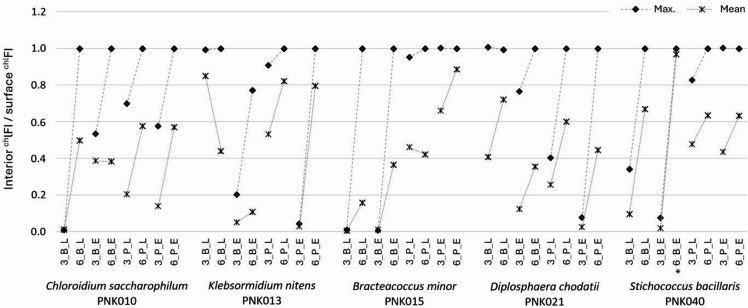
The rate of the chlorophyll fluorescence intensity (^chl^FI) between the interior and the surface of substrates detected at cross-sections of materials in the confocal scanning-laser microscope after three (3) and six (6) months of algal cultivation on brick (B) and plaster (P) in laboratory (L) and environmental (E) conditions; (*) point at sample with p-value > 0.05 in the interior and surface ^chl^FI, while dash line connects the change between the 3^rd^ and 6^th^ months of the experiment.

The changes in ^chl^FI between the surface and interior parts of the substrates were statistically significant (*p* ≤ 0.05), except for the *S. bacillaris* biofilm on brick in the environment after 6 months of cultivation (*p* = 0.404) (Fig. 5, Supplementary Table S8). After three months of cultivation and biofilm adaptation to experimental substrates, a high level of chlorophyll fluorescence quenching (^chl^FI_q_) – exceeding 92% was recorded for the biofilms: *C. saccharophilum* on brick in the laboratory, *B. minor* on brick in both locations, *S. bacillaris* on brick in the environment, and both *K. nitens* and *D. chodatii* on plaster in the environment (Fig. 5, Supplementary Table S9).

In most cases, the mean rate of ^chl^FI between the interior and surface of substrates (int/sur ^chl^FI) increases with cultivation time, with the highest change between the 3^rd^ and 6^th^ months for *S. bacillaris* on brick in the laboratory (Δ^3–6^ int/sur ^chl^FI = 0.56). The decrease in the mean rate was recorded for *K. nitens* on a brick in the laboratory (Δ^3–6^ int/sur ^chl^FI = −0.41) and slightly for *B. minor* on a plaster in the laboratory (Δ^3–6^ int/sur ^chl^FI = −0.04) (Supplementary Table S9). Despite minor differences in the mean ^chl^FI value, the rate based on maximum ^chl^FI in almost all cases reveals equal ^chl^FI between the surface and interior of the substrate (max. int/sur ^chl^FI = 1.00). Only for *K. nitens* growing on brick in the environment after 6 months, the rate is slightly lower (max. int/sur ^chl^FI = 0.77) than in others (Fig. 5, Supplementary Table S9).

After six months, CLSM revealed distinct algal fluorescence across all substrate cross-sections, with markedly stronger interior colonisation observed in plaster (Supplementary Figs. S11–S15).

**Fig. 6:**
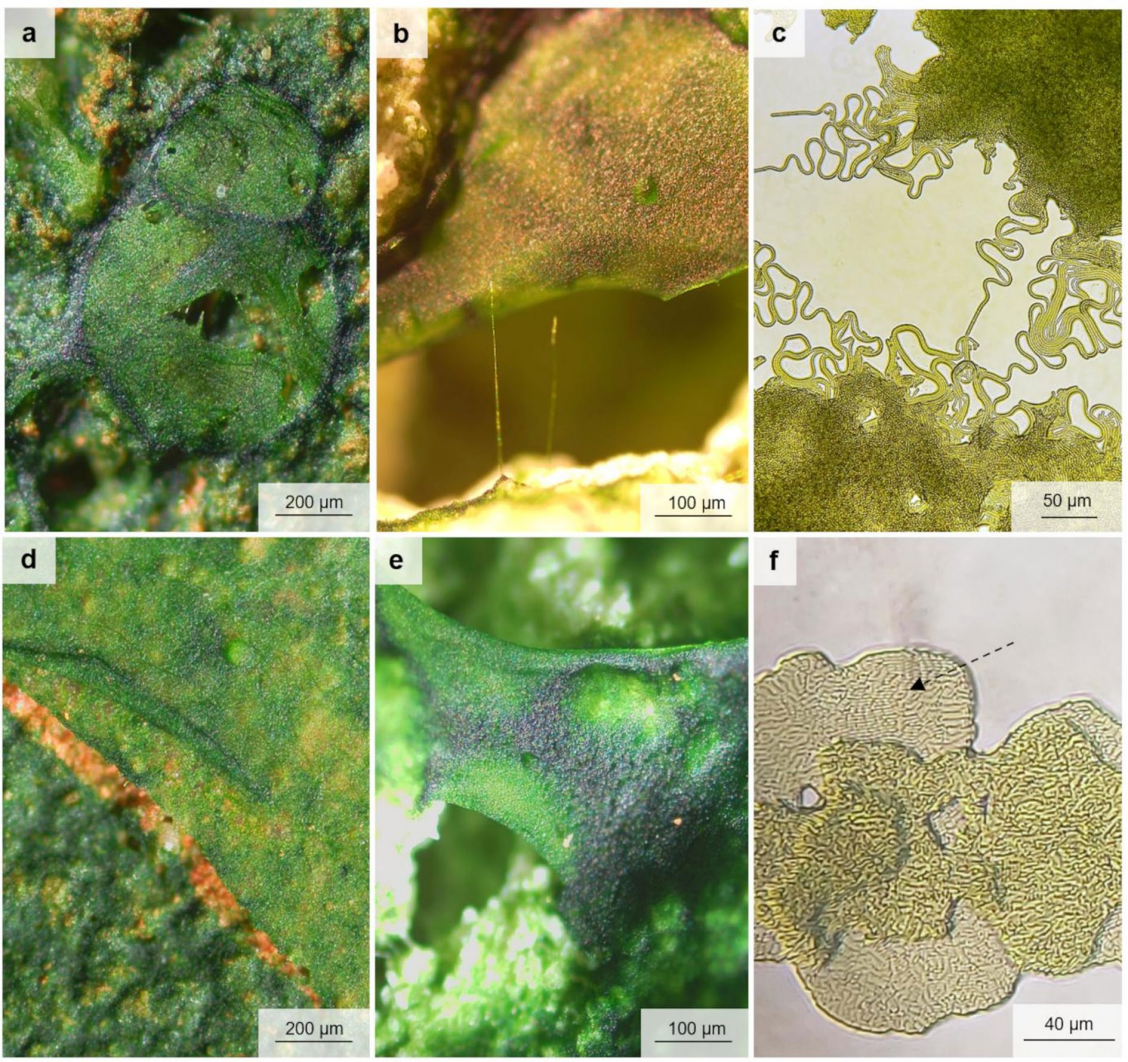
Images of (**a–c**) the *Klebsormidium nitens* PNK013 biofilm on (**a**) experimental brick, (**b**) experimental plaster, and (**c**) in agar culture, and (**d–f**) the *Stichococcus bacillaris* PNK040 biofilm on (**d**) experimental brick, (**e**) experimental plaster, and (**f**) in agar culture; arrow points at directional development in a part of the biofilm.

All algae in the coccal form after three months of cultivation exhibited more intense growth within the natural pores and cavities of both substrate types than at the substrate surface (Supplementary Figs. S6, S8–S10). With time, their biofilm filled the pores and overgrew the surface of the substrates, forming a dense, compact, and smooth biofilm in the case of *C. saccharophilum*, *D. chodatii*, and *S. bacillaris*, and a less dense and compact biofilm in *B. minor* (Supplementary Fig. S16). The filamentous *K. nitens* initially exhibited the same growth strategy on substrates; however, over time, it began to overlay the pores and cavities with a more or less thick, membrane-like layer composed of algal long and short filaments (Fig. 6a, b,c; Supplementary Fig. S16c, d). The same growth strategy was noticed in S. *bacillaris* biofilm. After six months of cultivation in some parts of the substrates, similar layers as in *K. nitens*, which overlay the pores or grow between surface asperities, were observed (Fig. 6d, e). Even though *S. bacillaris* is a coccal alga, its elongated cells in some parts of the biofilm may divide along the same axis, creating a flat-like membrane that expands in one direction (Fig. 6f; Supplementary Fig. S16:i).

### The impact of aerial algal colonisers on substrates

To track the changes in the substrate surface as a result of biofilm growth, the total height of the substrate profile (Rt) was measured using scanning electron microscopy (SEM).

**Fig. 7:**
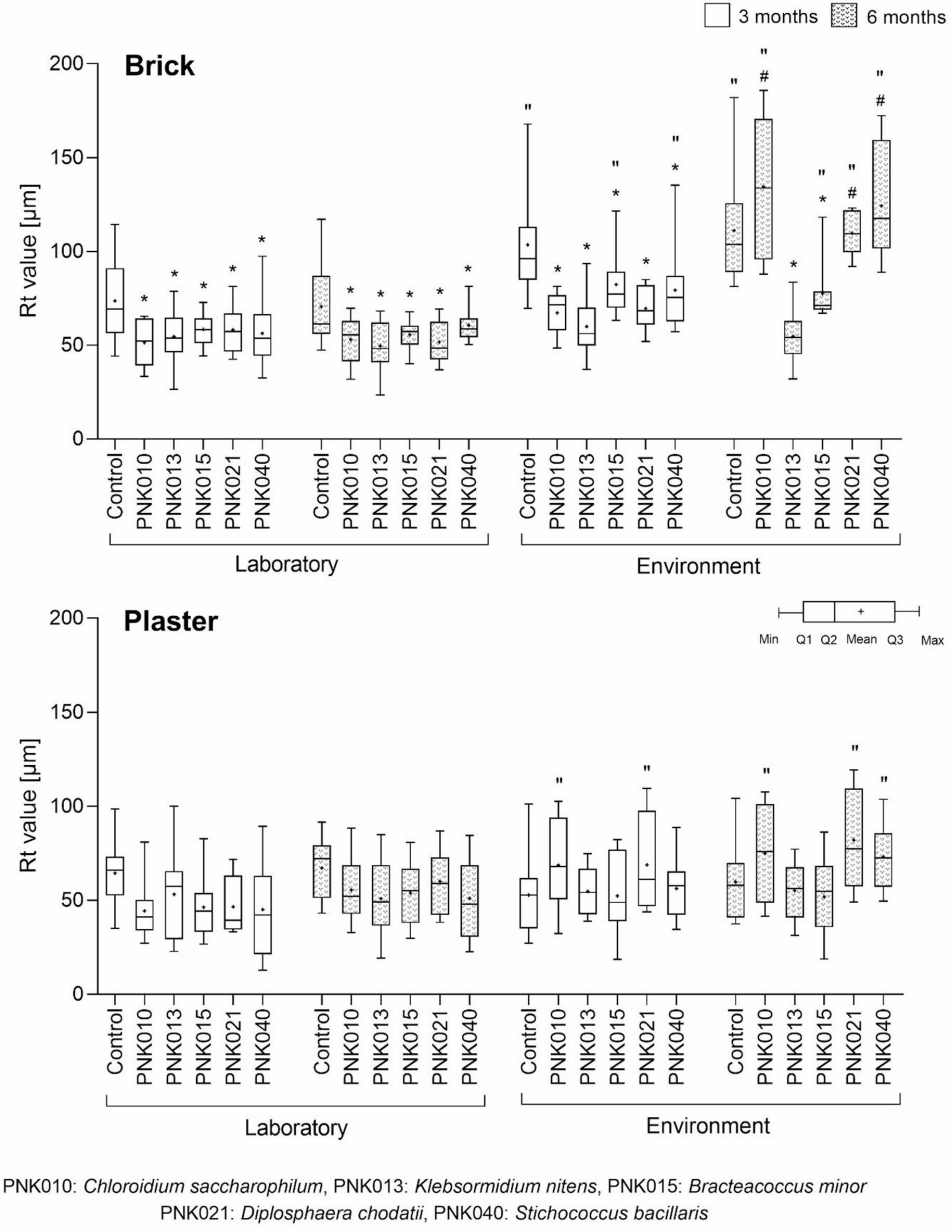
Changes in the total height of the substrate profile (Rt) (l = 300 μm) detected after three and six months of incubation on brick and plaster in the laboratory and environmental conditions, visualised by the box-and-whisker plot with (Q1–Q3) quartile set of data; statistical significance (q ≤ 0.05) is marked by: (*) in relation to control vs. PNK changes, (#) in relation to 3 vs. 6 month changes, and (“) in relation to laboratory vs. environment changes.

The range in the Rt of the control bricks changed only slightly with the experiment time (by 0.43 μm for the lab sample and 2.3 μm for the environmental sample) (Fig. 7), without any statistical significance (q > 0.05) (Supplementary Table S10). However, in relation to the location, the change was significant for both the three-month and six-month incubation (q = 0.02 for 3 months and q = 0.003 for 6 months) (Supplementary Table S11). In the laboratory, individual biofilms impacted the surface of a brick substrate, lowering the mean Rt by 25.4% ± 4.7% after three months of incubation and by 22.0% ± 7.8% after six months of incubation (Fig. 7, Supplementary Table S10). Within three months in the environment, all biofilms exhibited a significant decrease in Rt compared to the control, with the most pronounced reduction observed in the *K. nitens* biofilm (42% decrease, q < 0.0001). Another three months of incubation in the environment altered the surface of the bricks colonised by algae. Biofilms of *C. saccharophilum* and *S. bacillaris* caused an increase in the mean Rt of the control by 20.8% and 11.8%, respectively, and a further decrease was caused by *K. nitens* and *B. minor*, by 50.7% and 30.2%, respectively. Nevertheless, the statistical significance of the changes in relation to the control was noted only in the cases of *K. nitens* (q < 0.0001) and *B. minor* (q = 0.007) (Supplementary Table S11).

The range in the Rt of the control plaster changed only slightly by 15.2 μm for the lab sample and 7.3 μm for the environmental sample; however, for the plaster in the environment, the rage of Rt was higher than for lab sample (74.0 μm after 3 months, 66.7 μm after 6 months for environment sample and 63.6 μm after 3 months, 48.4 μm after 6 months for lab sample) (Fig. 7, Supplementary Table S10). Statistical analyses revealed that neither the Rt change during the time nor the Rt change in relation to the location of control samples was statistically significant (q > 0.05) (Fig. 7, Supplementary Table S11). Given the growth of algal biofilms, they did not significantly alter Rt relative to the control during the experiment (q-values of 0.058–0.404 for lab samples and 0.122–1.000 for environmental samples). The only significant change was observed for *C. saccharophilum* (q = 0.034 for 3 months, q = 0.049 for 6 months), *D. chodatii* (q = 0.005 for 3 months, q = 0.005 for 6 months), and *S. bacillaris* (q = 0.023 for 6 months) samples in relation to the location (Fig. 7, Supplementary Table S11).

**Fig. 8:**
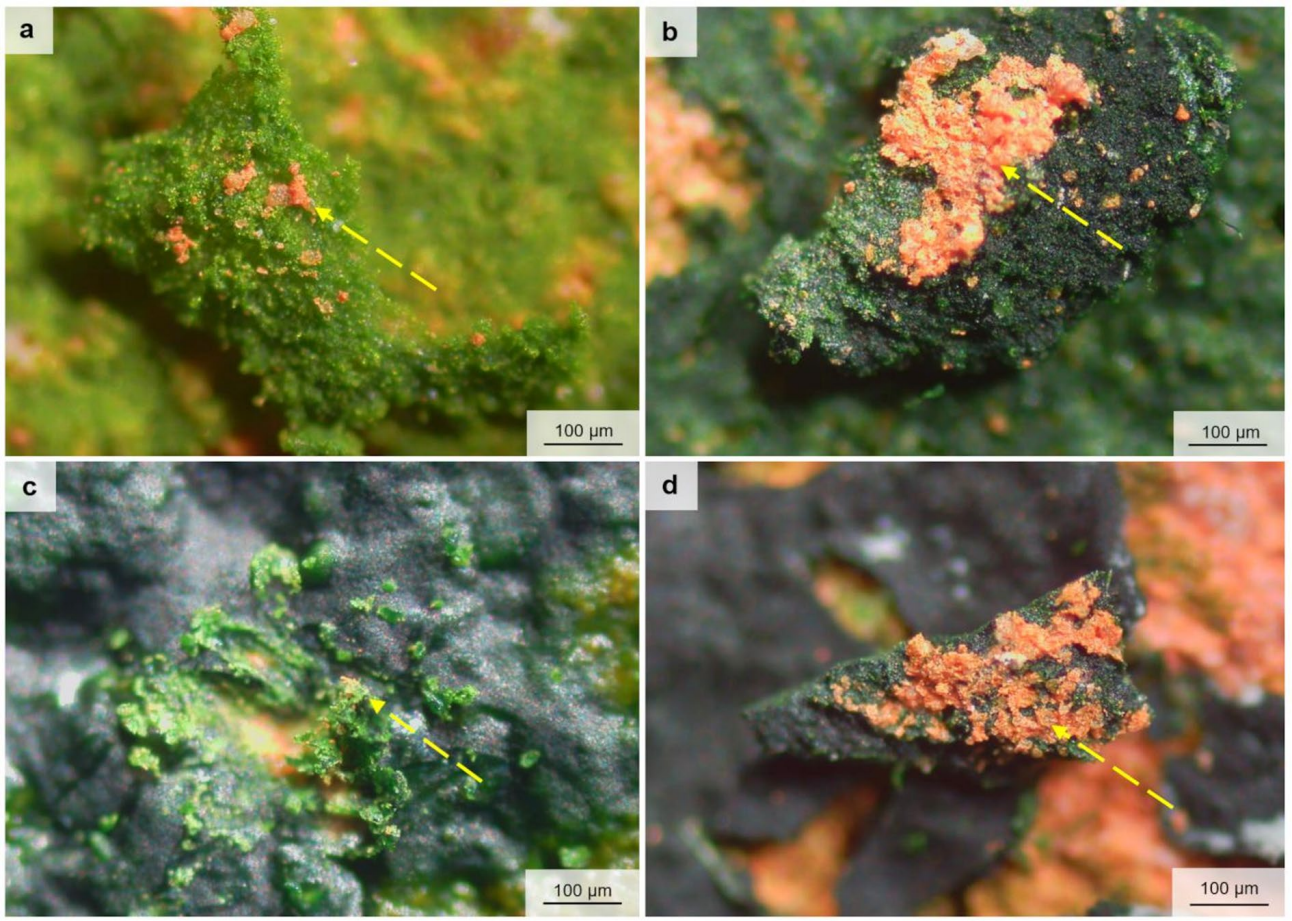
The biofilms sloughing from a brick surface with the surface part of the substrate, the detached mineral grains are pointed by dashed arrows (**a**) *Chloroidium saccharophilum* PNK010, (**b**) *Klebsormidium nitens* PNK013, (**c**) *Diplosphaera chodatii* PNK021, (**d**) *Stichococcus bacillaris* PNK040.

Microscopic observation of algal biofilms growing for six months on substrates revealed the presence of biofilm cracks and fractures (Supplementary Fig. S16). This phenomenon was observed in biofilms of *C. saccharophilum*, *K. nitens*, *D. chodatii*, and *S. bacillaris*. In some samples, the width of cracks and fissures reached up to 50 μm. Moreover, *in situ*, the cleavages of the biofilms lead to detachment of the biofilm in large sheets from the substrates. In the case of plaster, the biofilm sloughing did not impact the material’s mineral structure; it merely created a new space for further algal development. However, in relation to bricks, the biofilm detachment caused structural damage to the surface of the bricks. With the biofilm sloughing, the mineral grains of the brick surface were torn off, increasing the roughness of the substrate and exposing the inner layers. Such an impact was observed for the biofilms of *C. saccharophilum*, *D. chodatii*, and *S. bacillaris*, as well as the filamentous *K. nitens* (Fig. 8).

## Discussion

All the identified taxa may be found on different continents worldwide, including North America, Asia, and Europe^17–20^. Mostly, they were reportedly found in terrestrial habitats, but some, such as *C. saccharophilum*, *K. nitens*, and sporadically *B. minor*, were components of freshwater ecosystems^18,21,22^. Moreover, the *B. minor* and *C. saccharophilum* data on man-made surfaces also exist^18,23,24^. They are either free-living or, in some cases, such as *D. chodatii* and *C. saccharophilum*, they can form a lichen thallus with fungi^25–27^. Lichens exhibit significant biodeteriorative potential through the production of oxalic acid^3^. These oxalate deposits persist even after lichen death, contributing to ongoing deterioration. The role of algae in oxalate formation is merely indirect. They supply organic carbon to the fungal partner, which subsequently produces organic acids^28^; thus, they serve as a basis for lichen symbiont deteriorative metabolism^29^.

Although there are slight differences in the ecological preferences of the studied algae (i.e., between *K. nitens* and *B. minor* in terms of substrate humidity), their wide distribution allows to conclude that all of the identified taxa can tolerate a wide range of environmental conditions, and in the temperate climate zone, they are eurytypic.

In this research, after three months of adaptation, an intense biofilm growth was observed. The samples were then cultivated during the late summer/autumn season, which contributed to intensified growth after the adaptation period. Moreover, the overall algal biomass was higher on the bricks than on plaster, and the same tendency was reported by Komar et al.^16^. Variation in algal biofilm composition is primarily driven by seasonal fluctuations. Studies show that the communities recorded in spring and autumn possess many overlapping taxa^30^. The intense wind and rainfall events contribute to algal dispersion and an intense colonisation rate^31^. Therefore, both seasons may be crucial for accelerating deterioration, especially in brick, which is more susceptible to algal biofilm formation than plaster.

All five taxa in the first three months of growth filled the pores and microfractures, which can be considered a protective strategy for the biofilm, as the time after inoculation coincided with late spring and summer, when intensive isolation and desiccation impacted algal growth. Equal chlorophyll fluorescence at the surface and interior of the substrate confirms that, even after six months of development, the studied algae can survive and remain physiologically active within the material structure. The microorganism’s ability to grow within substrates involves two strategies: inhabiting existing rock spaces and/or actively boring into substrates by dissolving the substrate with terminal cells of endolithic filaments^32,33^. The latter mechanism is highly detrimental to the substrate’s structure and contributes to the material’s geophysical biodeterioration^3^. The mineral substrate provides crucial nutrients for growth, and even in its inner parts, a small amount of light, approximately 0.01% at a depth of 2.1 to 4.5 mm^34^, is still available for phototrophs. Moreover, the substrate pores and fissures protect inhabitants from ecological stressors and environmental changes^3,35^. The access of algal cells to the interior parts of the material was passive and enhanced by periodic sprinkling of lab substrates and by natural rainfall events in the environment. Even the filamentous *K. nitens* could freely colonise the pores as it easily disintegrates into short filaments and unicells that can pass through the pores with water deeper into the substrate.

The dense, compact biofilms smoothed the surfaces of the materials (in *K. nitens* and *S. bacillaris*, even forming a flat-like membrane that overgrew the pores) and narrowed the range of substrate profiles in both laboratory conditions and the environment. This is evident for all of the studied coccal algae, and in the case of filamentous *Klebsormidium*, it was already reported by Komar et al.^16^. Although the weather conditions *in situ* influenced the material structure of the control in such a way that the alterations done by *C. saccharophilum*, *D. chodatii* and *S. bacillaris* became less prominent, the biofilms of *B. minor* and *K. nitens* maintained this phenomenon. Such covering of the natural pores significantly alters the hygrothermal expansion, moisture retention, and thermal conductivity of substrates, leading to weakening of the structural matrix and increased geophysical biodeterioration^3^. By investigating the growth strategy of the studied algae step by step, it is found that it differs slightly from the literature description at the beginning of colonisation. Studies state that biofilm development induces surface erosion and increases the roughness and porosity of materials^3,35,36^; however, this phenomenon was observed with time.

On the brick surface, all algae with a thick cell wall (*C. saccharophilum*, *D. chodatii*, and *S. bacillaris*) and *K. nitens* tear off the substrate surface layer. There is a positive correlation between EPS production and cell wall thickness in microorganisms^37^. After six months of development, not only were the biofilms so dense that their mass incorporated in the attachment of mineral grains to the biofilm, but the production of EPS was so intense that the substrate parts were firmly bound to the biofilms. Then changes in biofilm water content led to cracks and cleavages, which, under wind, were detached from the substrate, tearing apart mineral grains of the brick. The phenomenon of material loss due to crust removal has been observed in both field studies and under laboratory conditions^6^. Still, it has not yet been recorded or visualised in particular green algal species. In the end, such biofilm-substrate loss increases the roughness of materials; however, this was noted only in the case of brick.

As the results show, greater algal biomass occurred on brick substrates than on plaster, and biofilms smoothed the bricks’ natural pores and ultimately contributed to substrate mineral loss. Both substrates exhibit different properties, not only in porosity but also in chemical composition, which determines pH, water absorption, retention capacity, surface temperature, and nutrient availability. Bricks are composed mainly of silicates and aluminosilicates, with a relatively neutral to slightly alkaline surface, while plasters, due to calcium hydroxide content, are typically strongly alkaline. High alkalinity can inhibit the germination and growth of some algal species, at least temporarily, until surface carbonation lowers the pH over time^12,38,39^. Bricks have a high water absorption coefficient^40^, enabling the retention of moisture necessary for algal metabolism and photosynthesis. On the other hand, plaster may either rapidly absorb water and dry out quickly or resist moisture penetration altogether^41^, thereby influencing how long algal biofilms can persist. The thermal mass of brick allows it to retain heat longer than plaster, which can accelerate evaporation^42^ and limit algal survival. Still, this effect applies only to sun-exposed conditions. Dust accumulation within brick pores can provide micronutrients that facilitate algal colonisation, while plaster generally offers lower nutrient availability^43^, which can delay colonisation. All these differences explain the higher colonisation rate of biofilms at brick substrates.

The *B. minor* was the only species that was not involved in substantial deterioration of substrates. Its biofilms were less compact, and after six months of development, neither cracks, fissures, nor peeling of the brick substrate were observed. The formation of its green biofilm contributed only to the aesthetic deterioration of materials. Moreover, such deterioration by *B. minor* was observed in 1963 at the important historical site, the Lascaux Cave^44^. Its biofilms were responsible for the mass aesthetic degradation of paintings in the cave, leading to the cave’s closure to visitors.

This study shows how particular species` biofilms impact the mineral substrates at an early stage of colonisation. In the environment, algal biofilm biodiversity is highly significant. They act as complex microecosystems in which functional complementarity and synergistic interactions between phototrophs and heterotrophs are evident. Their synergistic metabolism enhances weathering reactions, moisture retention, and mechanical stress, all of which contribute to the long-term degradation of building materials^45–47^.

In conclusion, *C. saccharophilum*, *B. minor*, *D. chodatii*, *S. bacillaris*, and *K. nitens* are eurytypic aerial algae capable of colonising mineral substrates, showing greater growth on bricks than plaster. After an adaptation, they form biofilms of varying density, with *C. saccharophilum* emerging as the most vigorous coloniser. These species not only cause aesthetic deterioration but also contribute to geophysical degradation by filling and overgrowing the natural pores. Four species (*C. saccharophilum*, *K. nitens*, *D. chodatii*, and *S. bacillaris*) directly affected the mineral structure of bricks, promoting extensive biodeterioration. These algae represent important detrimental factors in the biodeterioration of brick substrates.

These findings emphasize the need to consider early algal colonisation in conservation strategies, as it can initiate long-term damage to architectural materials. By providing a framework for assessing species-specific mechanisms on material integrity, this work contributes to a more comprehensive understanding of how early-stage algal colonisation influences the long-term durability of built heritage and contemporary architectural materials alike. Addressing these biological processes is vital not only for protecting man-made objects but also for maintaining the cultural and aesthetic value of historic environments that underpin the tourism sector. Future research will focus on the metabolic and geochemical contributions of these taxa, determining whether they produce reactive or hazardous metabolites that compromise the chemical stability of substrates.

## Materials and methods

### Algal biofilms survey

Algal material was collected from brick and plaster walls of a residential tenement building in Łódź City (Poland), in an area of dense urban development, located far from city green zones. Sampling sites (*n* = 20) were selected based on the presence of green biofilm at an early stage of formation. For the study, only ‘young’ biofilms with uniform structure, barely visible as greenish staining, were selected, as this may indicate the beginning of colonisation by pioneer algae. Biofilms were collected during several days of the same week, every time at the same hour of a day (10.00–11.00 am), using a sterile soft brush onto the agar plates with BBM medium^48^ and cultivated for one week in laboratory conditions for the algal adaptation to *ex situ* conditions (in a growth chamber equipped with shelves providing individual but uniform lighting: artificial light from fluorescent tubular lights of 40 µmol/m^2^/s in a 16 h/8 h day/night period, stable temperature 20°C/15°C day/night temperature cycle and air humidity of 60% ± 5%). On the sampling day, air and substrate temperatures, as well as the air humidity and substrate moisture, were measured using an Elmetron PWT-411 field hygrometer (Elmetron, Warsaw, Poland) and a Testo 606-2 material moisture meter (Testo, Pruszków, Poland). Moreover, the light intensity at the biofilm spot was measured using an Abatronic AB-8809 A field lux meter (Mera, Warsaw, Poland) and expressed as a percentage of the illuminance at the biofilm development site compared to the open air as a reference (background). All environmental characteristics are in Supplementary Table S1. From biofilm samples, different algal morphotypes were isolated using a Nikon Eclipse Ts2 inverted optical microscope (Precoptic Co., Warsaw, Poland) to obtain unialgal isolates, which were further cultivated under the lab conditions described above.

### Characterisation of algal strains

After 60 days of cultivation, the morphology of isolates was examined using a Nikon Eclipse 50i Light Microscope with DIC optics (Precoptic Co., Warsaw, Poland) and an OPTA-Tech digital documentation system. The individual morphotype group was characterised based on its morphometrics (cell diameter, length, width, and L/W ratio; *n* = 100; Supplementary Table S3), ultrastructure, and chloroplast shape. Chloroplast shape and structure were examined using a Leica TCS SP8 Confocal Laser Scanning Microscope (Leica Microsystems, Wetzlar, Germany) in the Laboratory of Microscopic Imaging and Specialized Laboratory Techniques, University of Lodz. The fluorescence excitation of the chlorophyll pigments was induced using a White Light Laser of 488 nm, while the detection was recorded in red at 650–750 nm. For the visualisation of lipids, the BODIPY^™^ 505/515 fluorescent dye (Invitrogen, Carlsbad, CA, USA) was used^49^. The fluorescence excitation was induced using a 405 nm diode, while the detection was recorded in green at 510–550 nm. The 3D microimages of cell content were made using LAS-AF 3.3.0.10134 software. The ultrastructure of cells were visualised and documented using a JEM 1010 transmission electron microscope (JEOL Ltd, Japan) at 80 kV. The preparation of slides for TEM observations was performed as follows. The algae were scraped from Petri dishes and fixed with 2.5% glutaraldehyde in 0.1 M cacodylate buffer, pH 7.15, for 3 h at 4°C. The samples were then centrifuged at 4000 rpm for 5 min, and rinsed with cacodylate buffer. After subsequent centrifugation, samples were resuspended in molten 2% agar. Agar embedding was performed to allow for the uniform distribution of material, enabling it to be processed as easily handled blocks. The agar blocks were then rinsed two more times with cacodylate buffer and postfixed in 1% osmium tetroxide for 3 h at 4°C. Subsequently, the material was dehydrated in 10%, 30%, and 50% ethanol, three times for 15 min. Samples were stained with a 50% ethanol solution of uranyl acetate for one night at 4°C. After further dehydration in graded ethanol (70, 80, 90 and 100%) and propylene oxide, the material was infiltrated and embedded in Epon-Spurr’s resin mixture. After polymerisation at 37, 60 and 72°C for 24 h, resin blocks were sectioned with a diamond knife using Ultra Cut E (Reichert Jung, Germany) ultramicrotome. Ultrathin Sects. (60–70 nm) were placed on formvar-coated nickel grids and stained with uranyl acetate and lead citrate for 20 min.

### Molecular and ecological identification of strains

The total DNA was extracted from the strains using either the DNeasy Plant Mini Kit (Qiagen, USA) according to the manufacturer’s protocol or, for samples where the kit-based isolation was insufficient, the Chelex 100 resin (Bio-Rad, California, USA) with the procedure as follows: 20 µl of cultures was centrifuged to obtain the cell’s pellet and resuspended in 10% of Chelex in Tris-HCl 10 nm (pH = 7.8). For proper denaturation and lysis of the cell-wall proteins, 5 µl of Proteinase K (20 µg/ml, Eurx, Poland) was added to the samples. The solution was vortexed and incubated in a thermoplastic shaker initially at 56°C for 45 min (with three vortexing cycles) and then at 98 °C for 30 min (without vortexing).

The identification of strains was based on nuclear (18S-ITS) and chloroplast (*rbc*L) DNA markers. The first marker was amplified as two overlapping regions using the following pair of primers 20F/18L^50^ and AL1500af/HLR3R^51,52^; while the *rbc*L marker was amplified using the PrasF/EllaR pair^53^. In the case of filamentous *Klebsormidium*, the nuclear marker was amplified as a single amplicon using a pair of primers NS7m/LR1850^54^. Primer sequences and PCR reaction conditions are in Supplementary Table S5. Amplicons were commercially cleaned and sequenced using Sanger sequencing with primers from PCR amplification at SEQme s.r.o. Company (Dobris, Czech Republic). The obtained sequences were assembled and cleaned using Geneious 11.1.5 software (Biomatters Aps, Aarhus, Denmark) and then compared with available sequences in the NCBI database using BLASTN^55^. For phylogenetic analyses, sequence data were aligned using MAFFT v. 7 web server^56^ and the phylogenetic calculations were performed using a Maximum likelihood analysis (ML) in IQ-TREE web server^57^ and Bayesian inference (BI) in MrBayes 3.2.2^58^. Phylogenetic trees were prepared in FigTree v. 1.4.3 software using the studied strains that resembled < 99.9% of the similarity matrix and NCBI data records set (Supplementary Table S4). For phylogeny, the best evolutionary models were calculated with the program Modeltest 3.7^59^ using the Akaike Information Criterion^60^. The settings of the best models are given in the legends of Supplementary Figs. S1–S4. The obtained sequences were submitted to the NCBI database under the following accession numbers: PX279515–PX279575 for the 18 S-ITS region and PX290146–PX290206 for the *rbc*L marker. Accession numbers for particular strains are in Supplementary Table S4. Raw data sequences, the alignment and tree files were also submitted to the figshare online database (10.6084/m9.figshare.30084811.v1).

The distribution of identified taxa in the native biofilms *in situ* was compared with the environmental data gathered during field surveys to characterise the variation in species occurrence and explained it by environmental factors important in algal colonisation. To select the best subset of factors for a multidimensional data analysis, an Interactive Forward Selection Test was performed. Data, which explained above 15% of the variation in species composition, were used in Canonical Correspondence Analysis (CCA)^61^. All calculations were done in Canoco for Windows 5.0 software (UCL, London, UK).

### Strains inoculation onto substrates and experiment design

The experiment was carried out on representative strains of the algal taxa – *Chloroidium saccharophilum* PNK010, *Klebsormidium nitens* PNK013, *Bracteacoccus minor* PNK015, *Diplosphaera chodatii* PNK021, and *Stichococcus bacillaris* PNK040. Strains were selected based on their molecular proximity to an epitype, high molecular similarity to other isolates, robust growth on agar plates, and consistent culture purity free of fungal contamination across cultivation cycles. The studied strains were suspended in BBM medium at an optical density of 1.00 and then transferred to clean, sterilised fragments of red brick and polysilicate plaster (experimental substrates) with a 4 cm × 4 cm working surface. Preparation of substrates was described in Komar et al.^16^. Following inoculation, the substrates were maintained in the laboratory under culture conditions, while some samples were placed in the environment for *in situ* experiments. Environmental samples were stored in an open space without any additional manipulation from spring to autumn. In contrast, the lab samples cultivated at the same time were sprayed every three weeks with 500 µl of sterilised tap water to simulate potential atmospheric impacts and to maintain proper humidity.

To record the first symptoms of potential substrate deterioration, after three and six months of the experiment, both algal growth and changes in the substrate structure resulting from cell division and biofilm formation were analysed.

### Characterisation of algal growth and its impact on substrates

The rate of algal growth was measured using chlorophyll a concentration (C_chla_). For the inoculation cultures (OD = 1.00) and the biofilms on experimental substrates after 1, 3, and 6 months of cultivation, spectrophotometric analysis of C_chla_ was performed using a Spectroquant Pharo 100 spectrophotometer (Merck, Darmstadt, Germany), according to the manufacturer’s protocol. All the samples were cultivated in three parallel repetitions; therefore, the measurements were made for three replicates of the samples (in total, for C_chla_ measurements, 108 pieces of bricks and the same for plaster were used). As the change in algal biomass between the 3^rd^ and 6^th^ months was high, a delta of the mean C_chla_ (ΔC_3–6_) was calculated.

Algal development on experimental substrates was visualised using a Nikon SMZ745T stereo microscope (Precoptic Co., Warsaw, Poland). For more accurate observations, an optical microscope (mentioned earlier) was used with external lighting. Moreover, surface SEM imaging was performed using a Phenom Pro-X Scanning Electron Microscope (Thermo Fisher Scientific, Waltham, MA, USA).

To examine the potential of algae to penetrate substrates, cross-sections of the substrates were prepared using a diamond steel knife and visualised using a Leica LSI Confocal Laser Scanning Microscope (Leica Microsystems, Wetzlar, Germany) in the Laboratory of Microscopic Imaging and Specialized Laboratory Techniques at the University of Lodz. The fluorescence excitation of the chlorophyll pigments was induced using a White Light Laser of 488 nm, while the detection was recorded in red at 620–750 nm. As the fluorescence intensity of chlorophyll pigments (^chl^FI) directly reflects the physiological state of phototrophs^62,63^, this variable was measured to assess the presence and photosynthetic activity of algal cells at the surface and the interior part of the substrate. All measurements were done in LAS-AF 3.3.0.10134 software in 180 imaging log replicates. For the correct inference of the algal penetration potential, the maximum and mean ^chl^FI quenching (^chl^FI_q_) between interior and surface biofilm fluorescence was calculated using Eq. (1) after 3 and 6 months of algal growth on substrates in situ and *ex situ* conditions:

1 $$\:{}_{.}{}^{\mathrm{c}\mathrm{h}\mathrm{l}}{\mathrm{F}\mathrm{I}}_{\mathrm{q}}\:\left[\mathrm{\%}\right]=100-\left(\frac{{}_{.}{}^{\mathrm{c}\mathrm{h}\mathrm{l}}\mathrm{F}\mathrm{I}\:\mathrm{i}\mathrm{n}\mathrm{t}.}{{}_{.}{}^{\mathrm{c}\mathrm{h}\mathrm{l}}\mathrm{F}\mathrm{I}\:\mathrm{s}\mathrm{u}\mathrm{r}.}\times\:100\right)$$

where: ^chl^FI_q_ – chlorophyll fluorescence intensity quenching [%]; ^chl^FI int. – chlorophyll fluorescence intensity in the interior part of the substrate; ^chl^FI sur. – chlorophyll fluorescence intensity at the surface of the substrate.

The distribution of ^chl^FI data was tested for normality using the Shapiro-Wilk test^64^. Since the data deviated from a normal distribution and were independent, a U Mann-Whitney test^65^ adjusted for tied ranks was used to assess the statistical significance of changes in ^chl^FI between the interior and surface parts of experimental substrates. The analysis was performed in PQstat 1.8.4 software (PQStat Software, Plewiska, Poland).

To assess whether biofilm formation alters the substrate surface structure, the roughness profile was analysed using the total height of the profile (Rt) value. The maximum profile peak height and the maximum profile valley depth along the 300 μm evaluation length at each sample were measured using the SEM microscope mentioned above and the Phenom ProSuite G6 software with the 3D Roughness Reconstruction module. For each sample, ten measurements were made at randomly selected sites after 3 and 6 months of algal growth on substrates *in situ* and *ex situ* conditions. The distribution of data was tested for normality using the Shapiro-Wilk test^64^. The data had a normal distribution and were independent; therefore, a one-way ANOVA for independent groups^66^ was chosen to analyse the statistical significance of the changes in Rt of experimental substrates. Since multiple variants were simultaneously tested, a two-stage linear step-up procedure of Benjamini, Krieger, and Yekutieli was used to control the false discovery rate. The method first estimates the proportion of true null hypotheses and then adjusts the significance threshold accordingly, giving the q-values (adjusted p-values)^67^. The statistical analysis and graphs were created using GraphPad 10.2.3. software (Dotmatics, Boston, MA, U.S.A).

## Supplementary Information

Below is the link to the electronic supplementary material.

Supplementary Material 1

Supplementary Material 2

## Data Availability

The datasets generated and analysed during the current study are available in the NCBI GenBank database [www.ncbi.nlm.nih.gov] under the accession numbers: PX279515–PX279575 for the 18S-ITS region and PX290146–PX290206 for the *rbc*L marker; the figshare database (https://doi.org/10.6084/m9.figshare.30084811.v1), and are included in this article (and its Supplementary Information files).
