# Supplementary material for: Growth strategy of aerial green algae on building materials in the temperate climate zone and its relevance to substrate biodeterioration
Source: Sci Rep. 2025 Dec 10;16:2167. doi: 10.1038/s41598-025-31926-x (PMC12808225; doi:10.1038/s41598-025-31926-x)
Supplement: Supplementary file 1 — Supplementary Material 1 [file 41598_2025_31926_MOESM1_ESM.pdf]

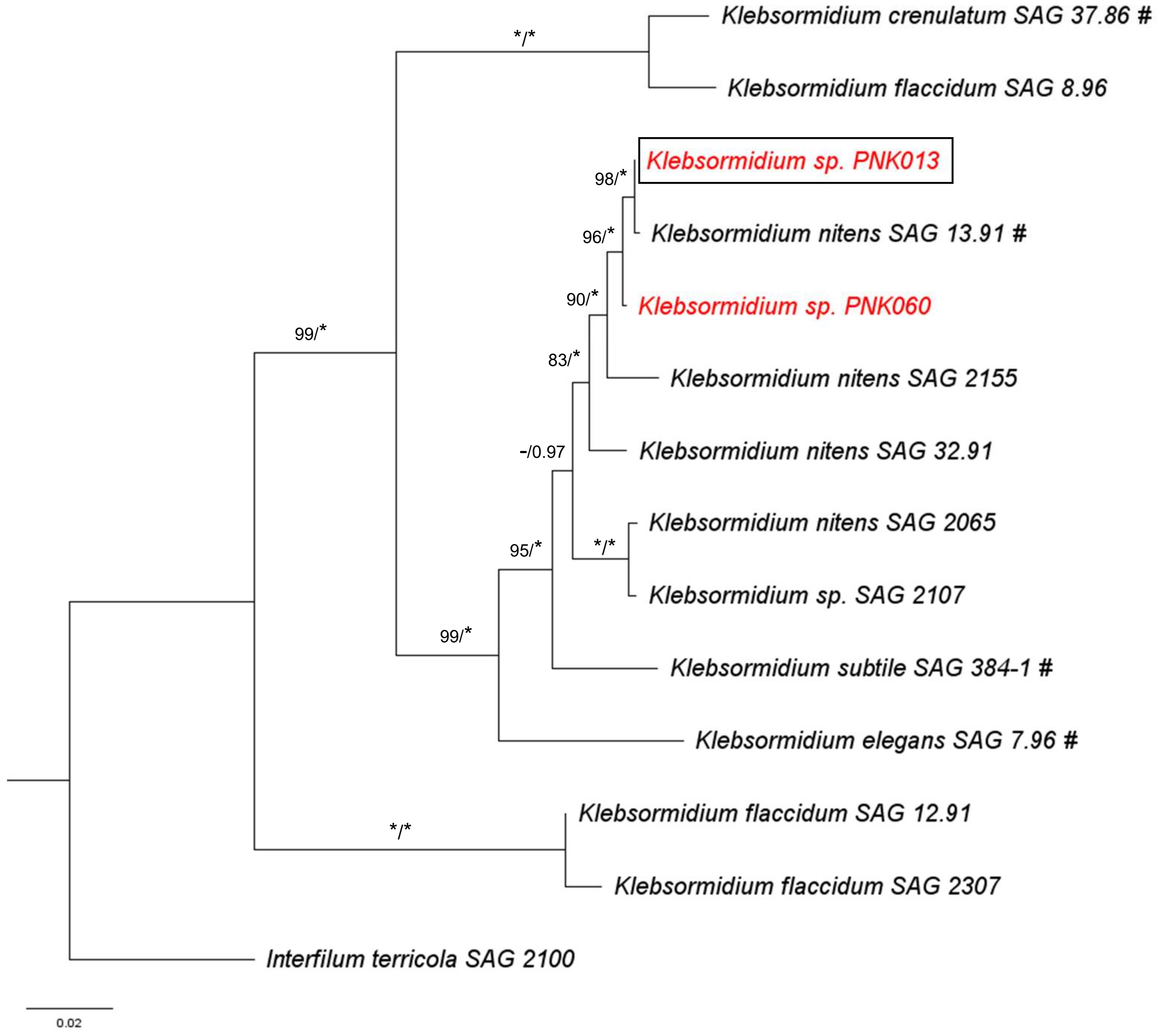

Figure S1. Molecular phylogeny of the *Klebsormidium* genus based on concatenated ITS26S*rbc*L matrix (1727 bp, ML topology of tree). The setting of the best evolutionary model is as follows: TIM2ef+G (rate matrix A–C 1.6177, A-G 3.5897, A-T 1.6177, C–G 1.0000, C-T 7.5511, G–T 1.000) with gamma shape parameter (G = 0.2760). Numbers on branches indicate bootstrap values from the Maximum Likelihood analysis, followed by posterior probabilities from the Bayesian Inference analysis. An asterisk (*) indicates 100 BS and 1.00 PP; a minus (-) value below 75 BS and 0.95 PP; a hash (#) indicates the epitype strains based on Mikhalyuk et al. (2015); while a box indicates the strain used for the experiment.

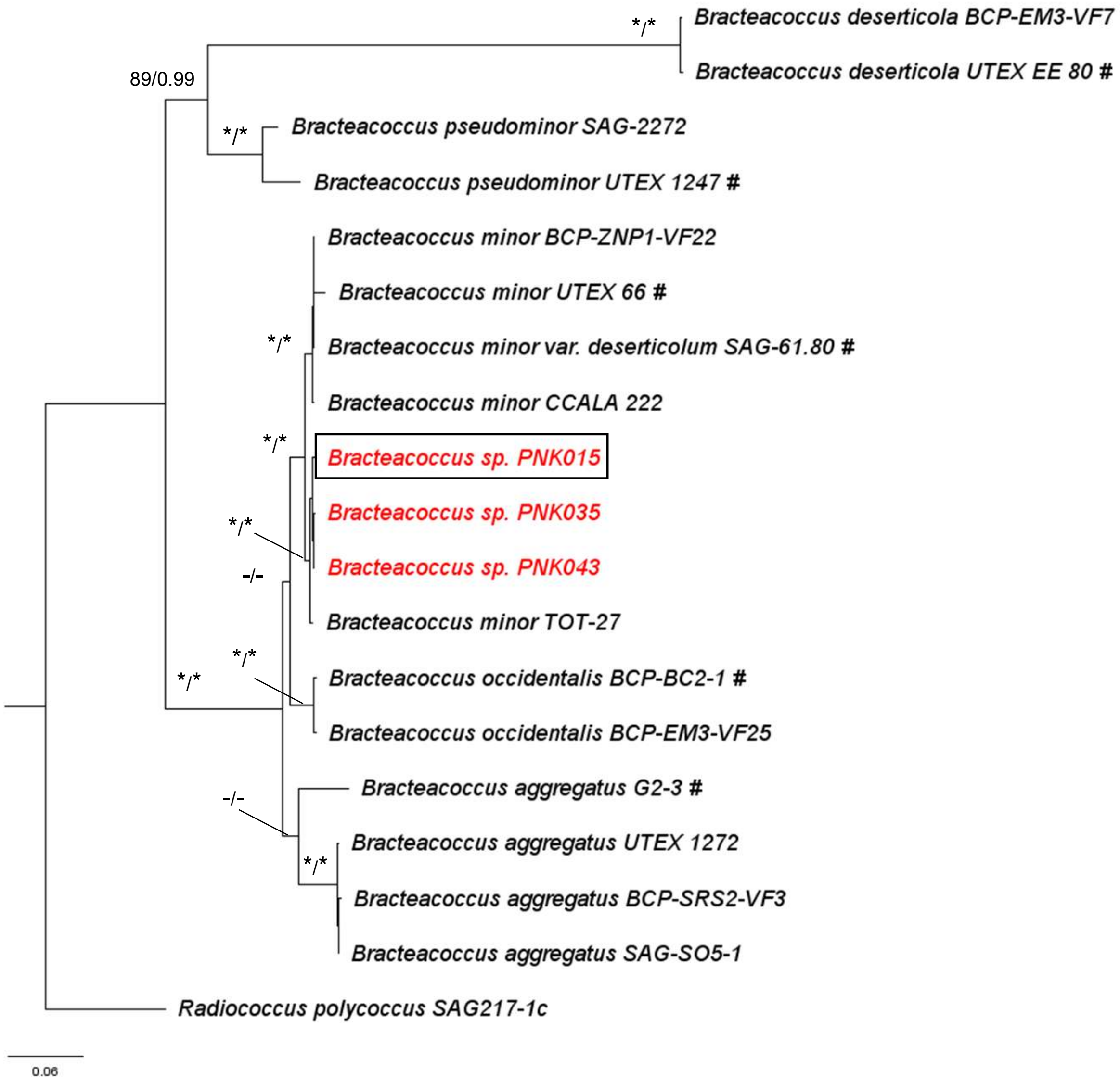

Figure S2. Molecular phylogeny of the *Bracteacoccus* genus based on concatenated 18SITS26S*rbc*L matrix (4368 bp, ML topology of tree). The setting of the best evolutionary model is as follows: TIM2+I+G (base frequencies: A 0.2459, C 0.2214, G 0.2656, T 0.2671; rate matrix A–C 1.3489, A-G 1.3422, A-T 1.3489, C–G 1.0000, C-T 2.2557, G–T 1.0000) with the proportion of invariable sites (I = 0.300) and gamma shape parameter (G = 0.5790). Numbers on branches indicate bootstrap values from the Maximum Likelihood analysis, followed by posterior probabilities from the Bayesian Inference analysis. An asterisk (*) indicates 100 BS and 1.00 PP; a minus (-) value below 75 BS and 0.95 PP; a hash (#) indicates the type strains based on Fučiková et al. (2012); while a box indicates the strain used for the experiment.

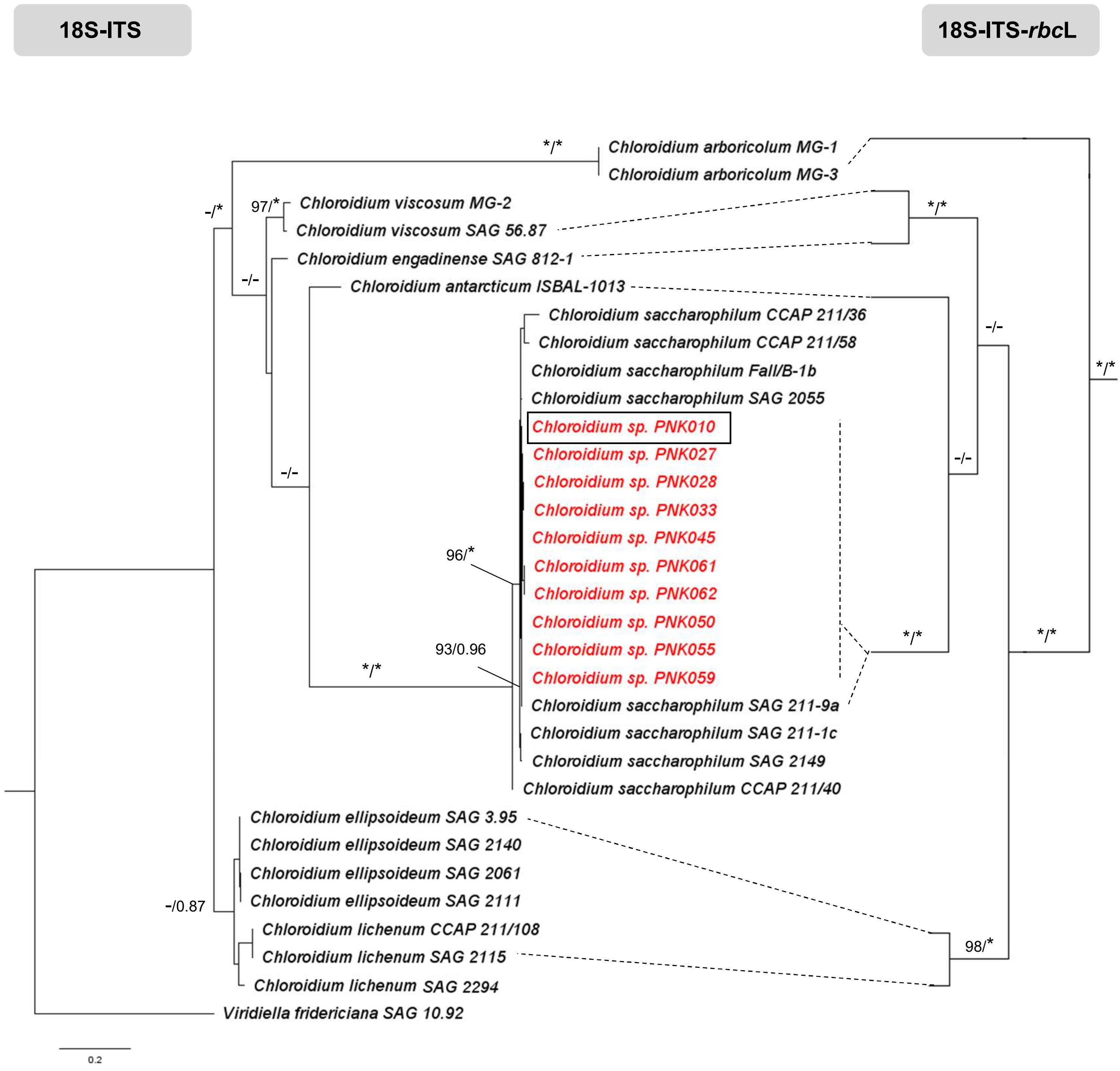

Figure S3. Molecular phylogeny of the *Chloroidium* genus based on concatenated 18SITS (3333 bp, ML topology of tree) and 18SITS-*rbc*L matrices (4456 bp, ML topology). The setting of the best evolutionary model is as follows: for 18S-ITS – TIM2+I+G (base frequencies: A 0.2214, C 0.2709, G 0.2992, T 0.2085; rate matrix A–C 1.1948, A-G 1.3819, A-T 1.1948, C–G 1.0000, C-T 2.4580, G–T 1.0000) with the proportion of invariable sites (I = 0.3240) and gamma shape parameter (G = 0.3650); for 18S-ITS-rbc*L* – TIM2+I+G (base frequencies: A 0.2408, C 0.2468, G 0.2755, T 0.2369; rate matrix A–C 1.3825, A-G 1.7298, A-T 1.3825, C–G 1.0000, C-T 3.1631, G–T 1.0000) with the proportion of invariable sites (I = 0.361) and gamma shape parameter (G = 0.413). Numbers on branches indicate bootstrap values (BS) from the Maximum Likelihood analysis, followed by posterior probabilities (PP) from the Bayesian Inference analysis. An asterisk (*) indicates 100 BS and 1.00 PP; a minus (-) value below 75 BS and 0.95 PP; while a box indicates the strain used for the experiment.

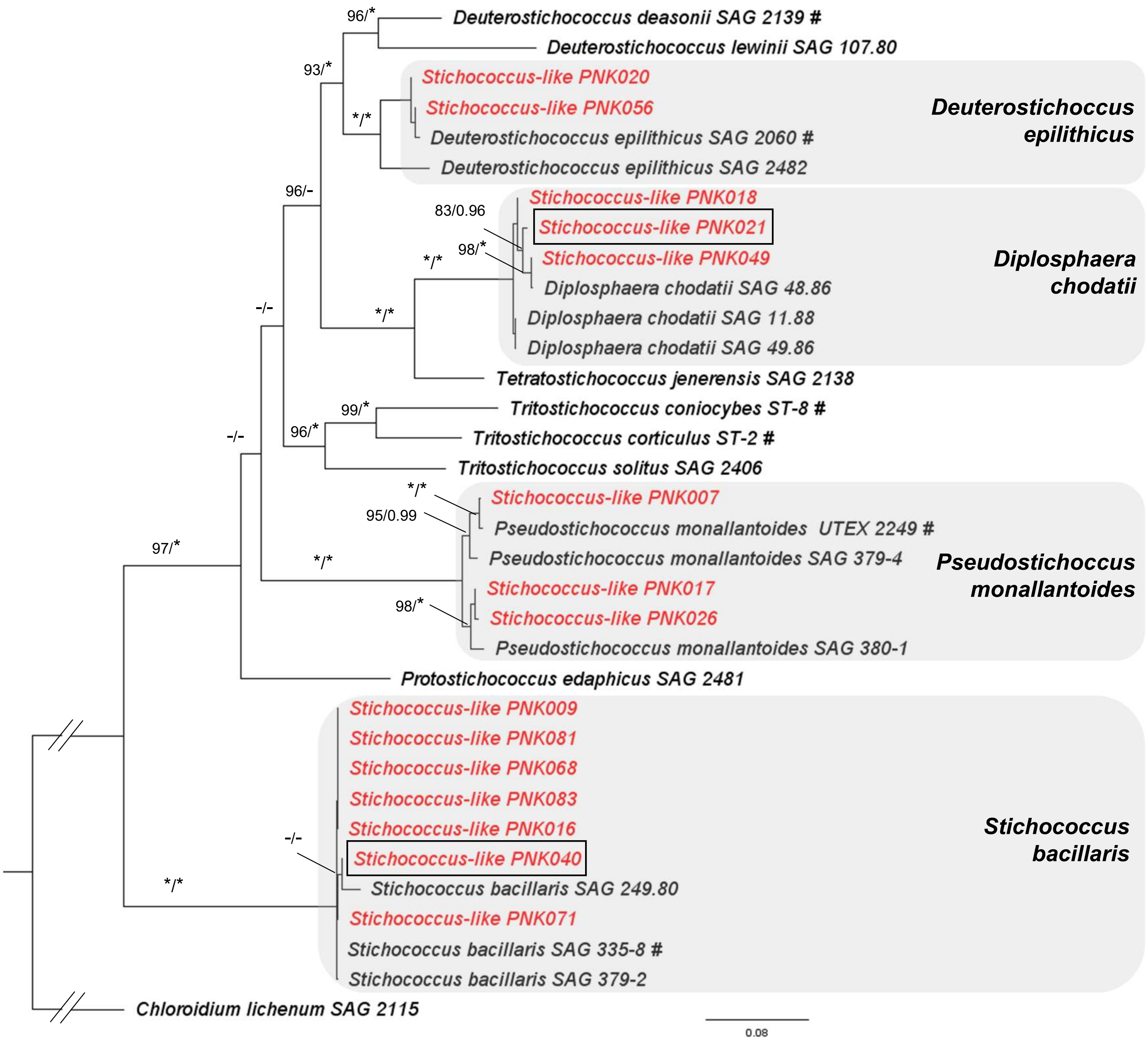

Figure S4. Molecular phylogeny of the Prasiola clade based on concatenated 18SITS26S*rbc*L matrix (6650 bp, ML topology of tree). The setting of the best evolutionary model is as follows: GTR+I+G (base frequencies: A 0.2479, C 0.2532, G 0.2654, T 0.2335; rate matrix A–C 1.3192, A-G 2.7361, A-T 2.1926, C–G 1.0724, C-T 6.0377, G–T 1.000) with the proportion of invariable sites (I = 0.437) and gamma shape parameter (G = 0.362). Numbers on branches indicate bootstrap values (BS) from the Maximum Likelihood analysis, followed by posterior probabilities (PP) from the Bayesian Inference analysis. An asterisk (*) indicates 100 BS and 1.00 PP; a minus (-) value below 75 BS and 0.95 PP; a hash (#) indicates the epitype and holotype strains based on Pröschold & Darienko (2020), while a box indicates the strain used for the experiment.

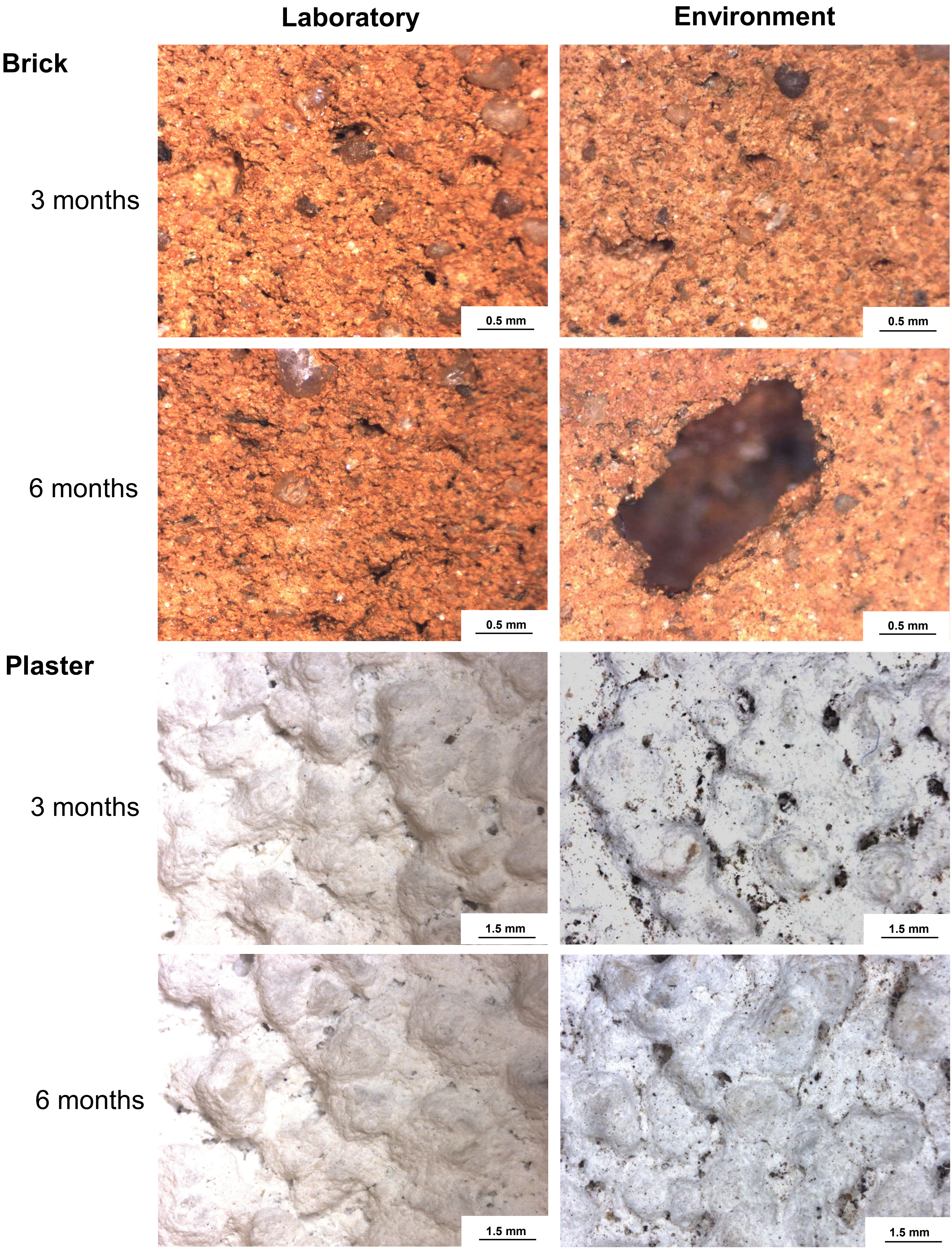

Figure S5. Control experimental substrates stored in the laboratory and environmental conditions for 3 and 6 months.

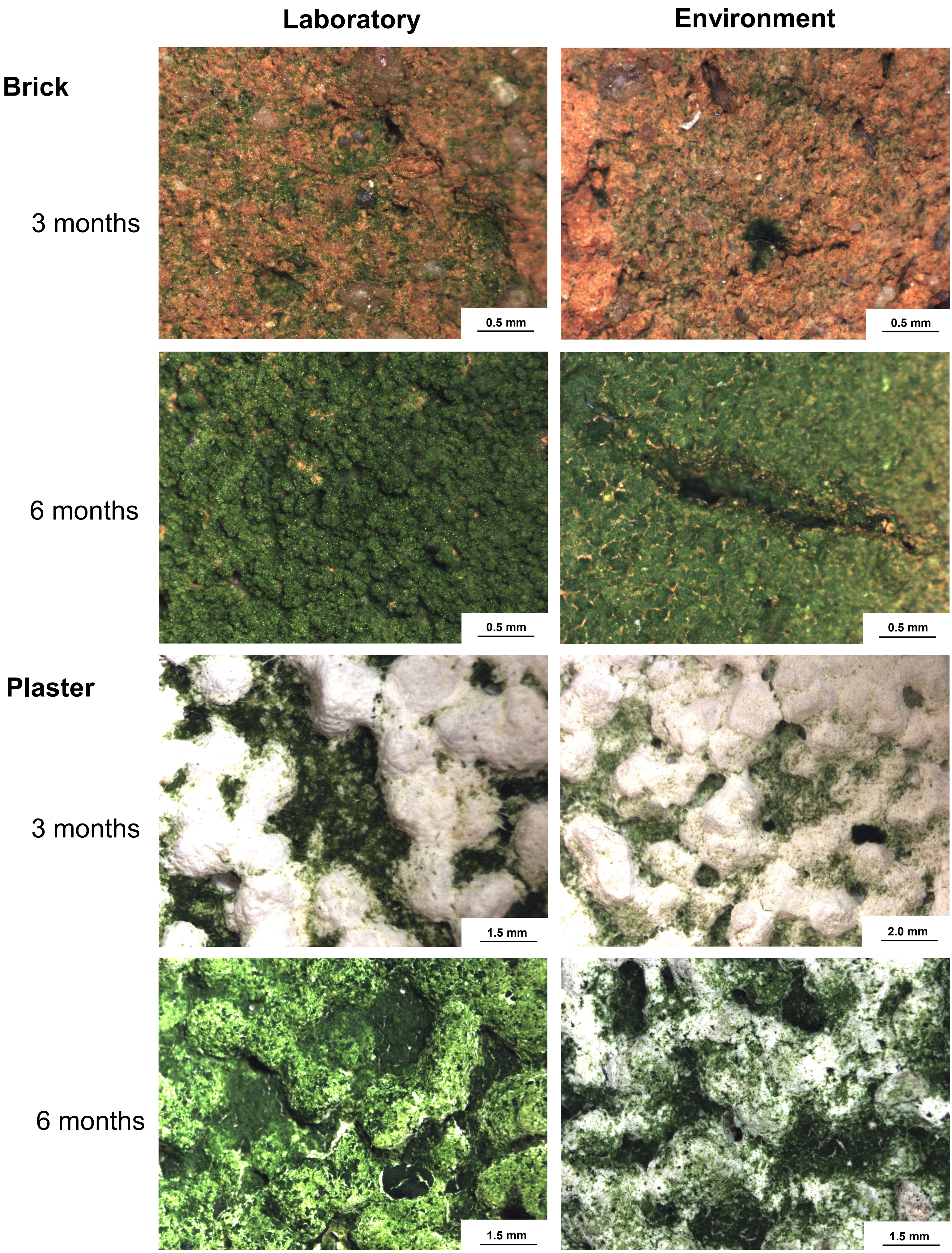

Figure S6. Biofilm development of *Chloroidium saccharophilum* PNK010 on experimental substrates in the laboratory and environmental conditions after 3 and 6 months of cultivation.

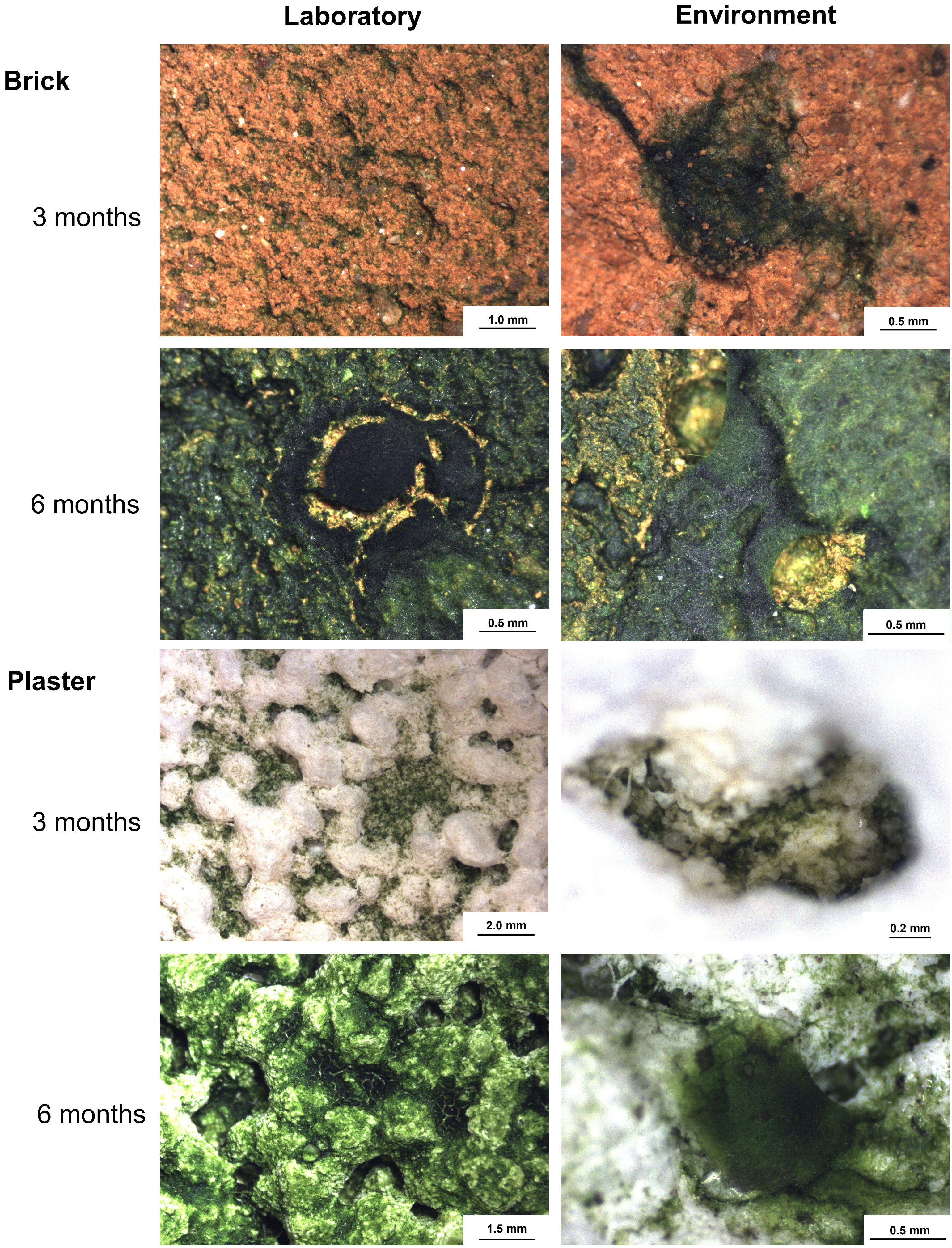

Figure S7. Biofilm development of *Klebsormidium nitens* PNK013 on experimental substrates in the laboratory and environmental conditions after 3 and 6 months of cultivation.

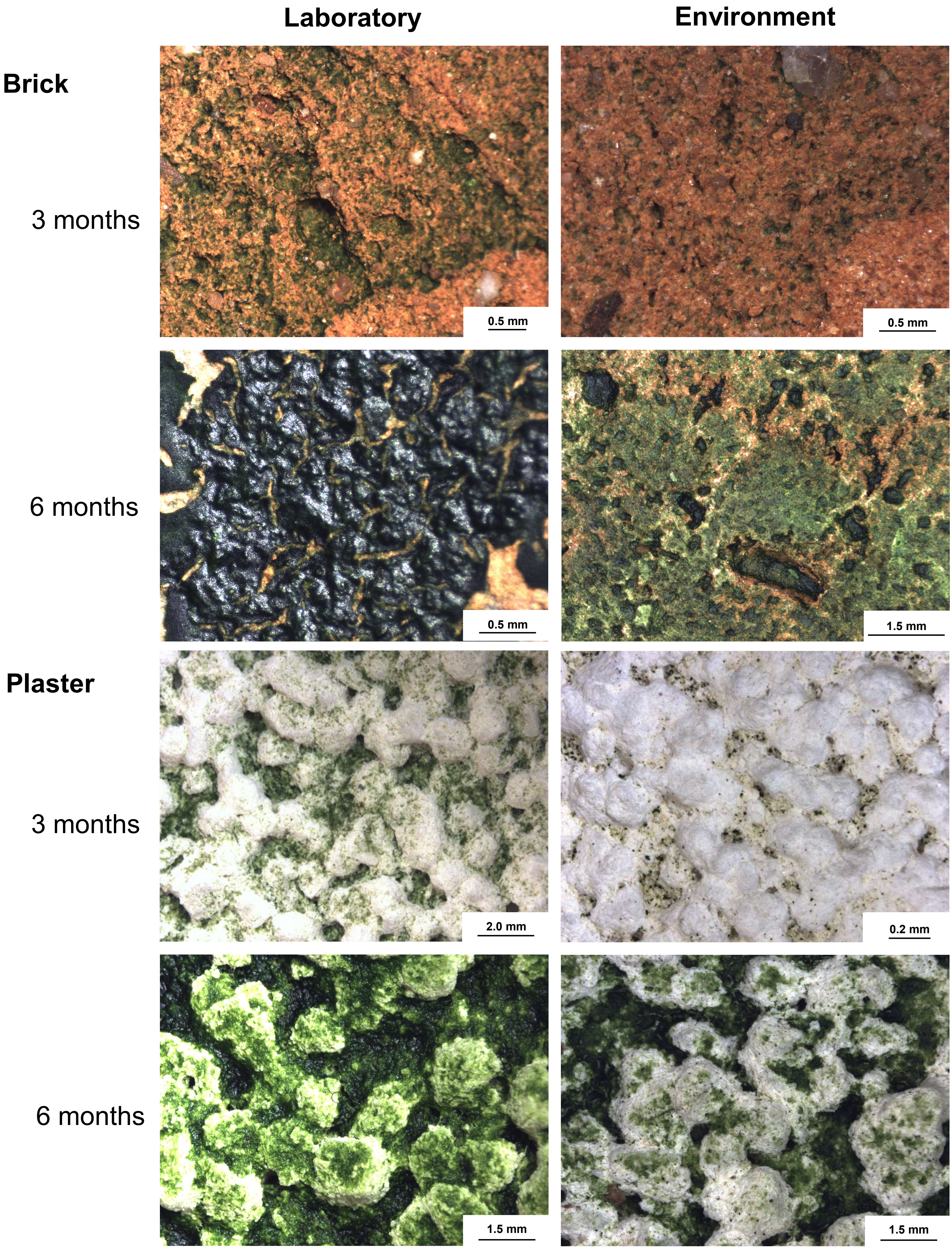

Figure S8. Biofilm development of *Bracteacoccus minor* PNK015 on experimental substrates in the laboratory and environmental conditions after 3 and 6 months of cultivation.

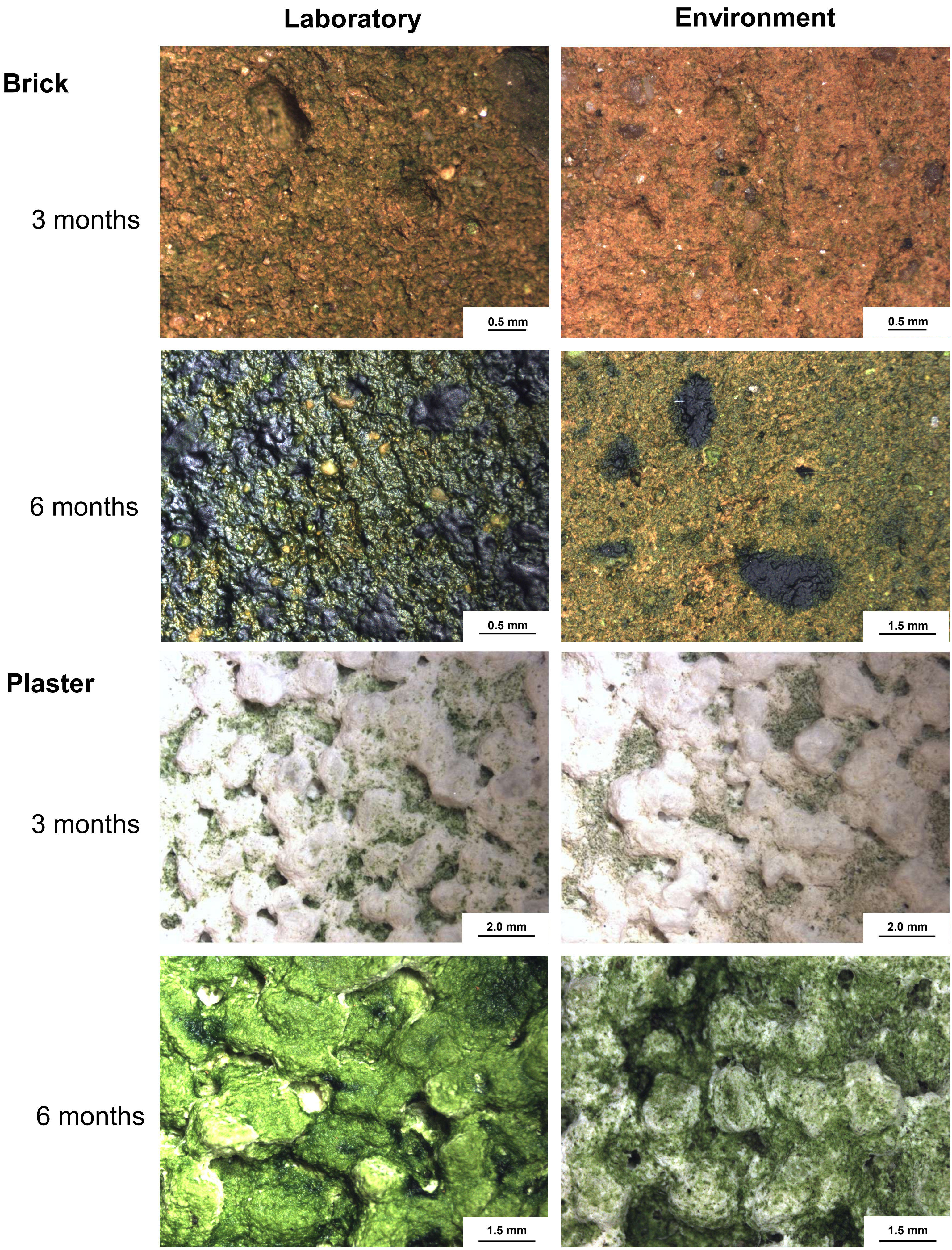

Figure S9. Biofilm development of *Diplosphaera chodatii* PNK021 on experimental substrates in the laboratory and environmental conditions after 3 and 6 months of cultivation.

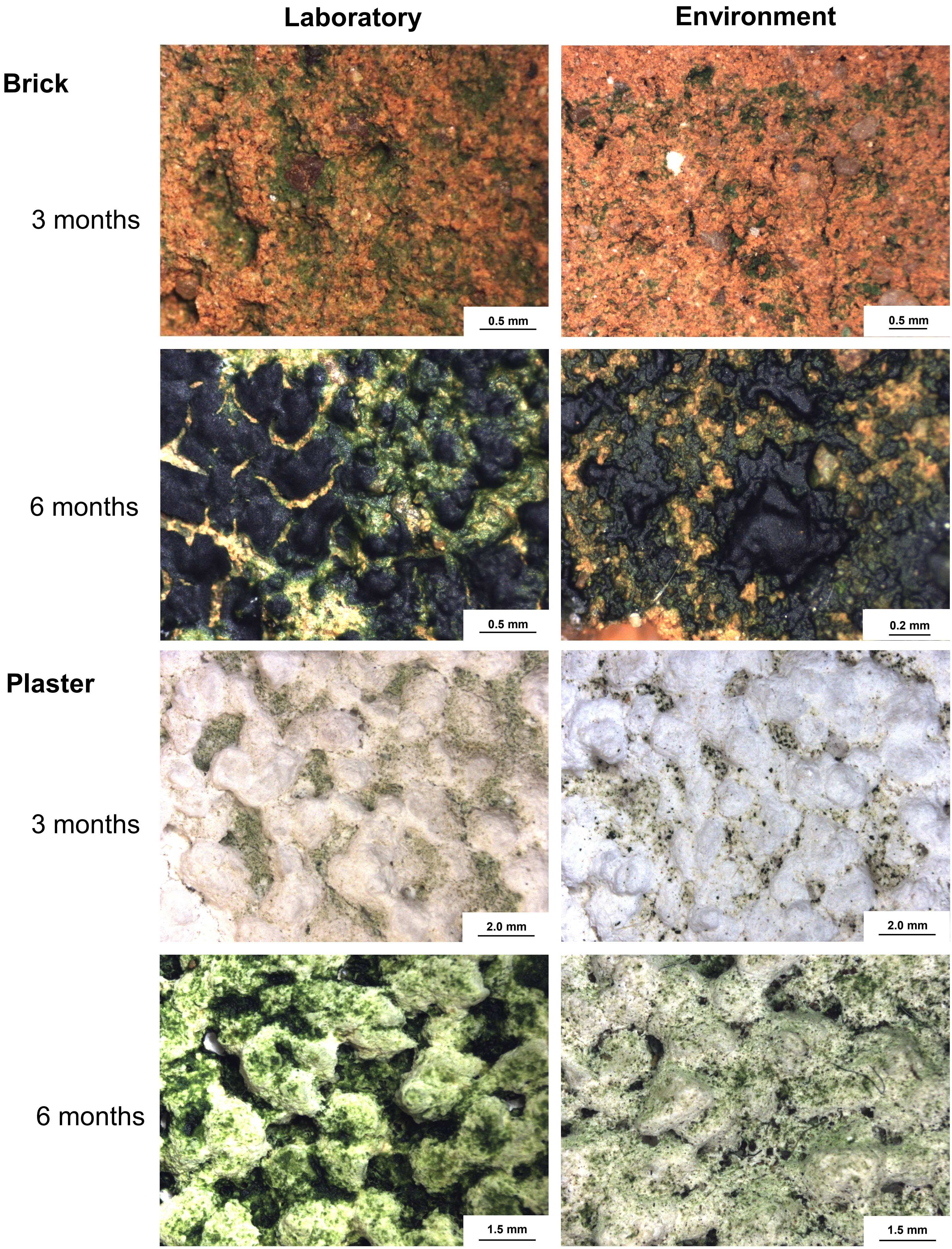

Figure S10. Biofilm development of *Stichococcus bacillaris* PNK040 on experimental substrates in the laboratory and environmental conditions after 3 and 6 months of cultivation.

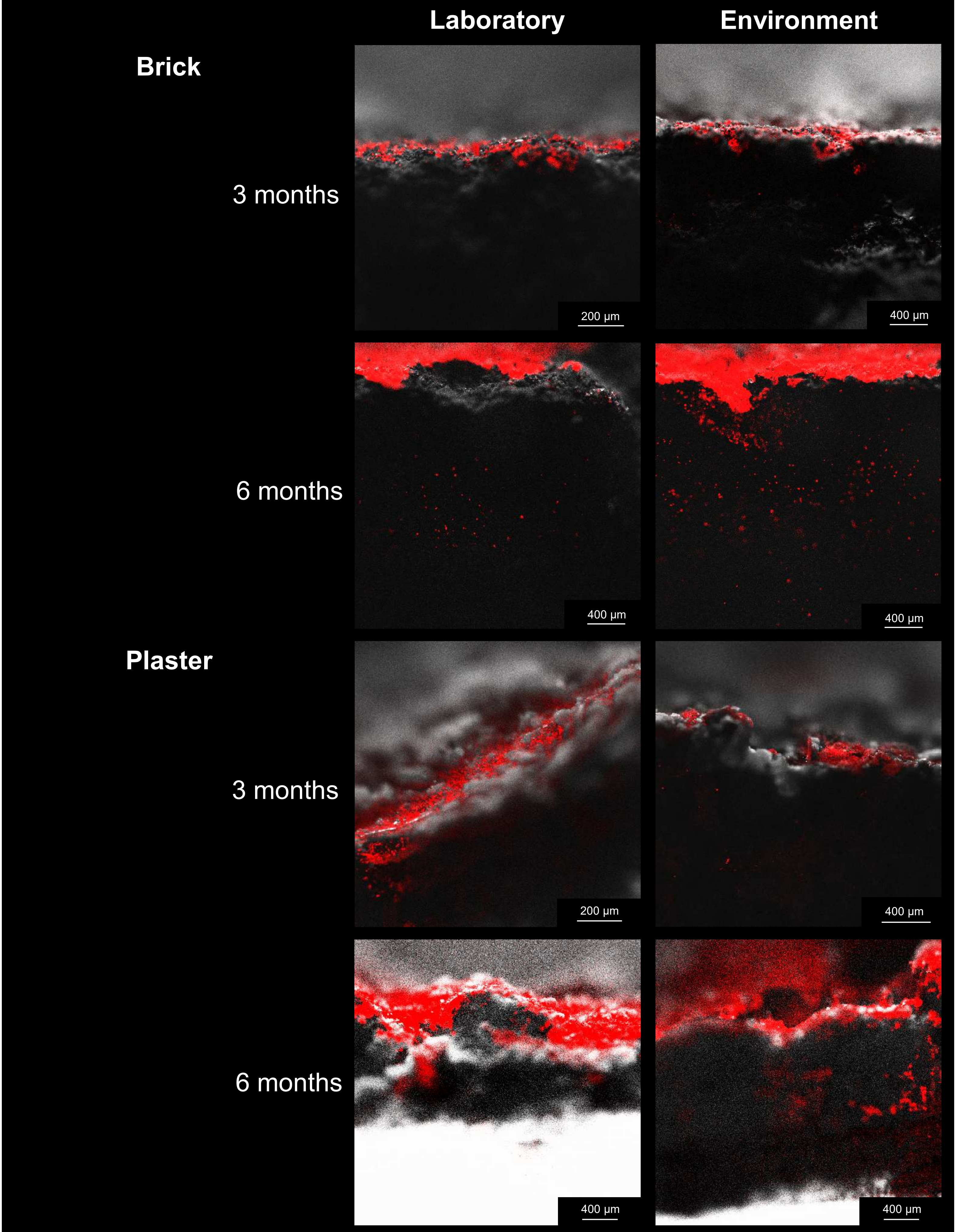

Figure S11. Biofilm fluorescence (in red) of *Chloroidium saccharophilum* PNK010 on a brick and plaster; a cross-sectional LSI CLSM view of substrates in the laboratory and environmental conditions after 3 and 6 months of cultivation.

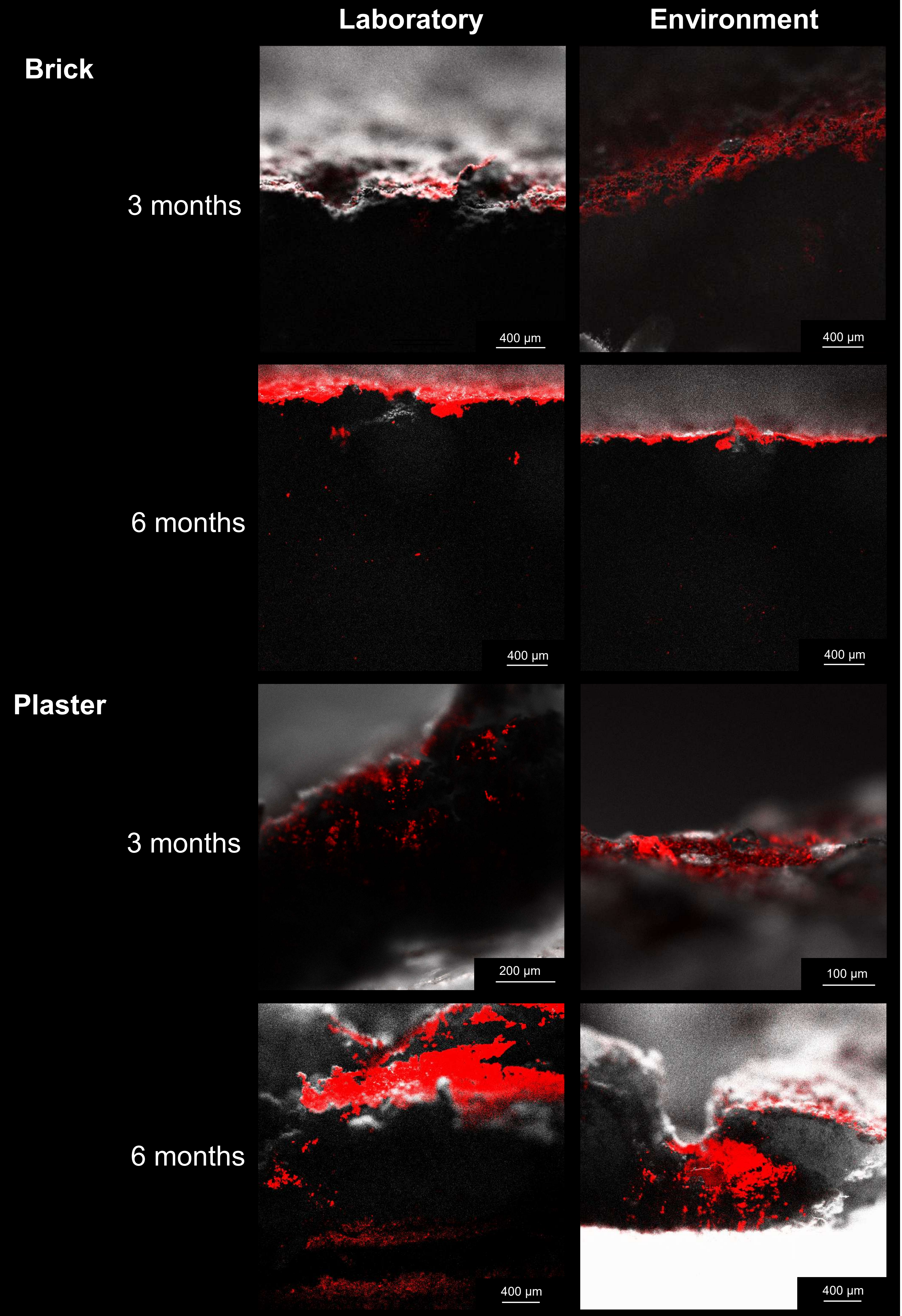

Figure S12. Biofilm fluorescence (in red) of *Klebsormidium nitens* PNK013 on a brick and plaster; a cross-sectional LSI CLSM view of substrates in the laboratory and environmental conditions after 3 and 6 months of cultivation.

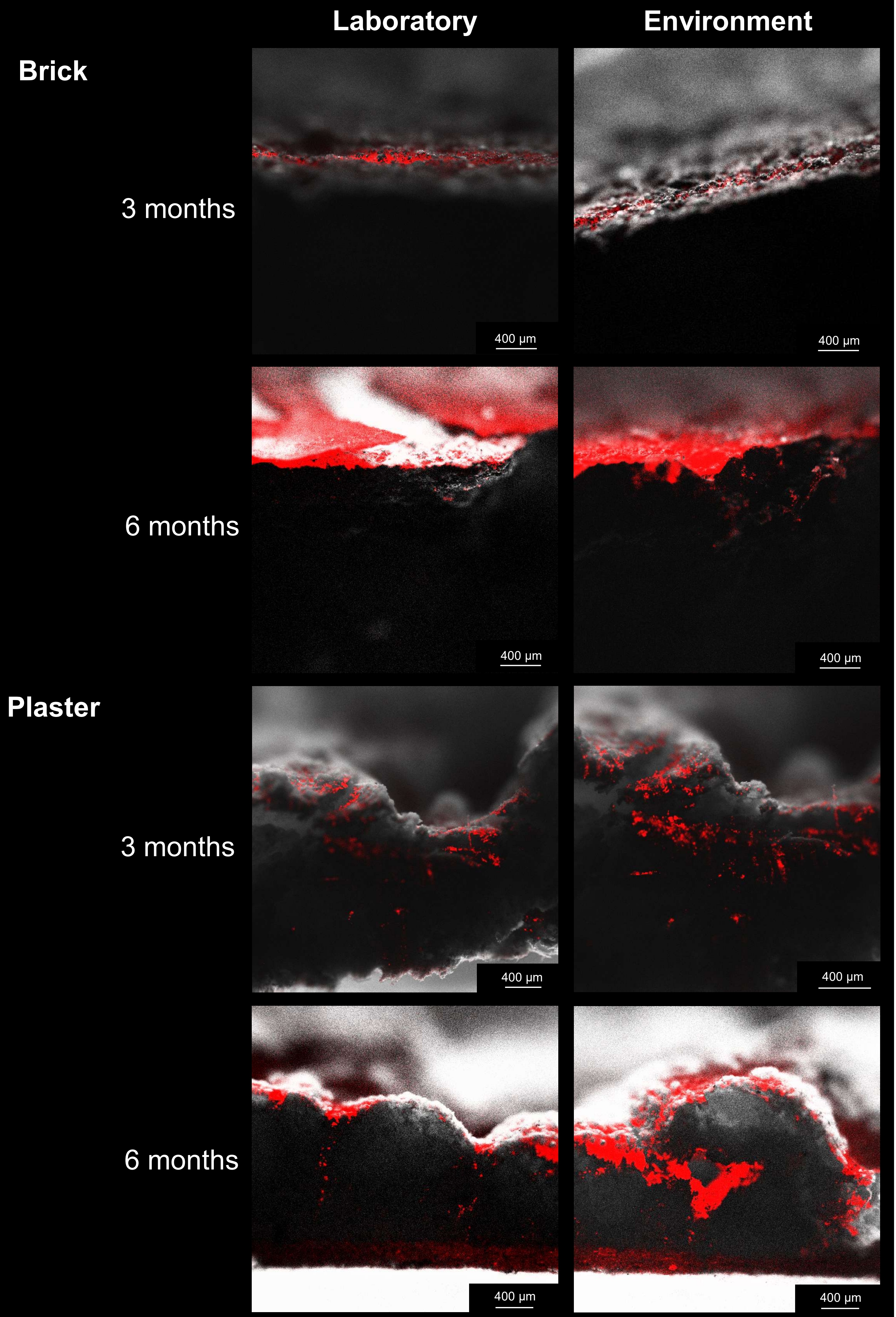

Figure S13. Biofilm fluorescence (in red) of *Bracteacoccus minor* PNK015 on a brick and plaster; a cross-sectional LSI CLSM view of substrates in the laboratory and environmental conditions after 3 and 6 months of cultivation.

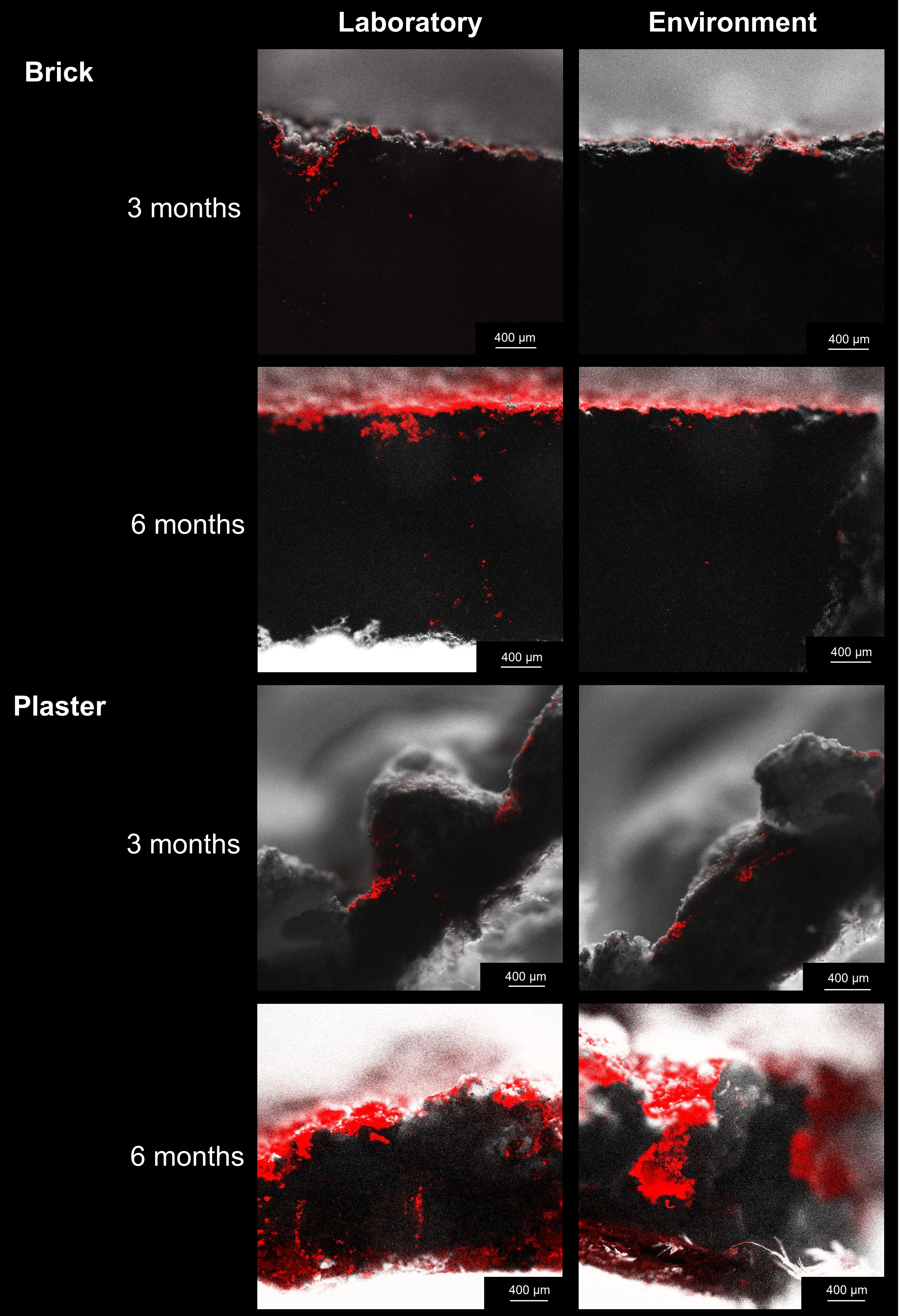

Figure S14. Biofilm fluorescence (in red) of *Diplosphaera chodatii* PNK021 on a brick and plaster; a cross-sectional LSI CLSM view of substrates in the laboratory and environmental conditions after 3 and 6 months of cultivation.

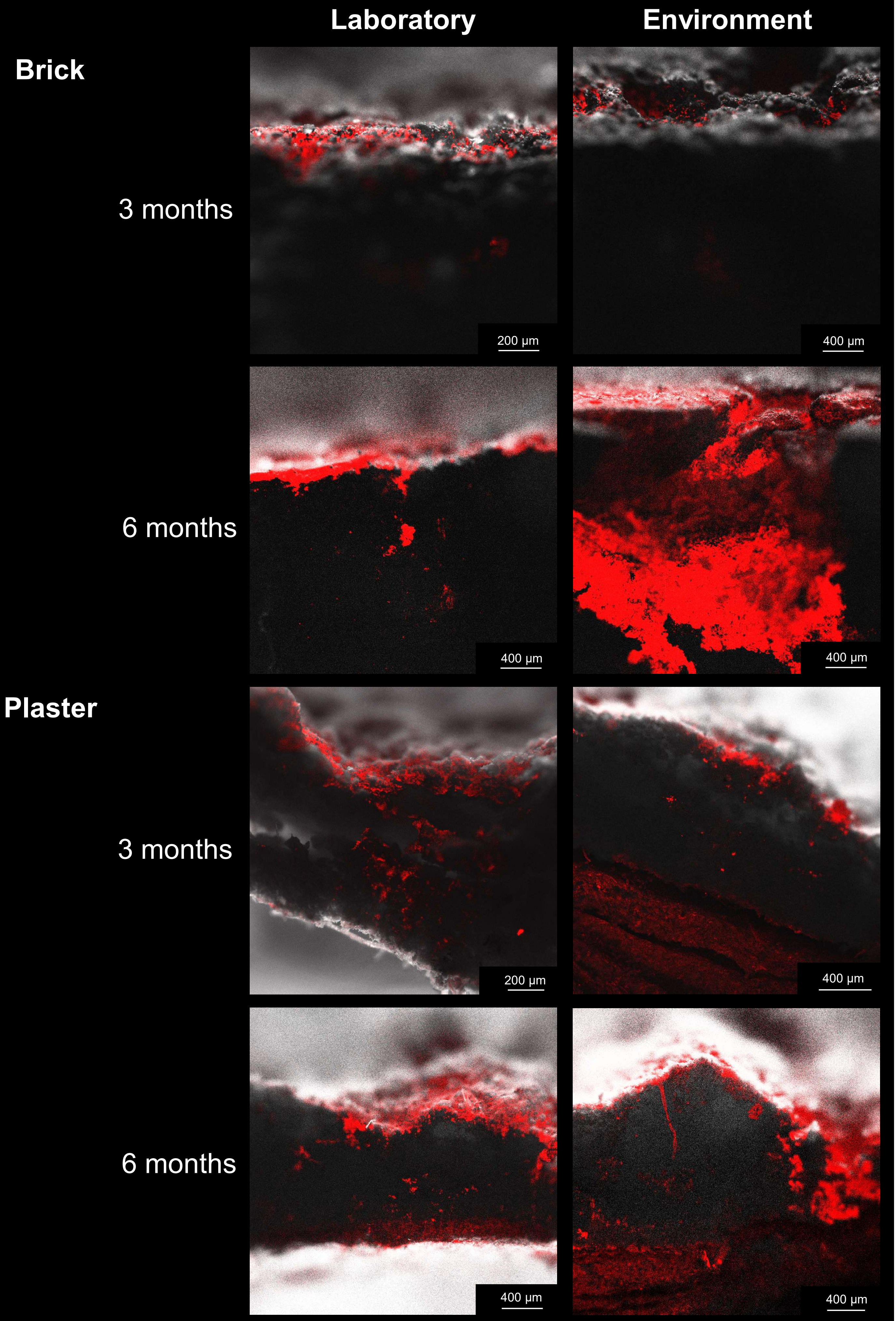

Figure S15. Biofilm fluorescence (in red) of *Stichococcus bacillaris* PNK040 on a brick and plaster; a cross-sectional LSI CLSM view of substrates in the laboratory and environmental conditions after 3 and 6 months of cultivation.

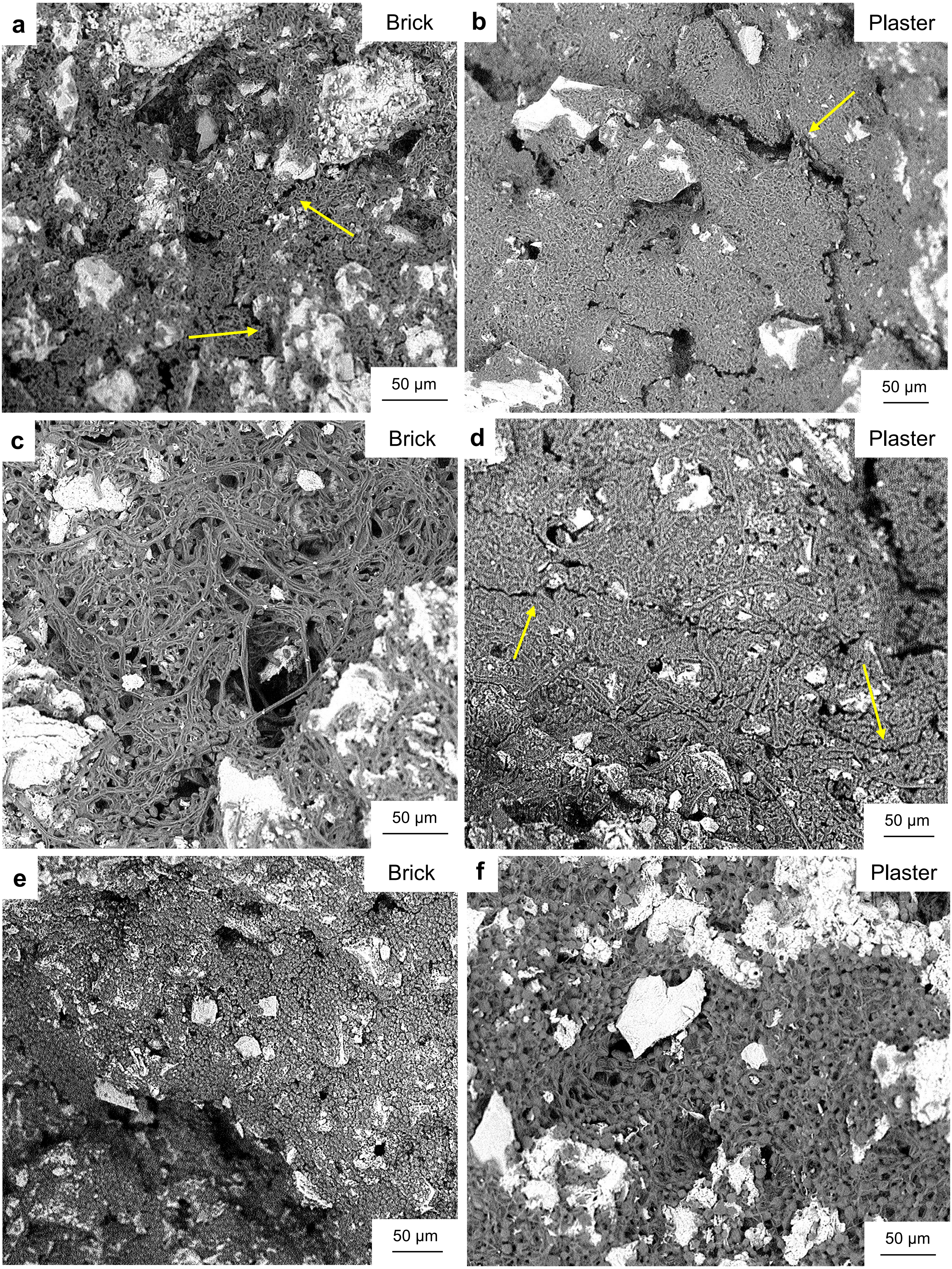

Figure S16. Algal biofilms in SEM on brick and plaster substrates; a-b. *Chloroidium saccharophilum* PNK010, c-d. *Klebsormidium nitens* PNK013, e-f. *Bracteacoccus minor* PNK015; solid arrows point at biofilm cracks.

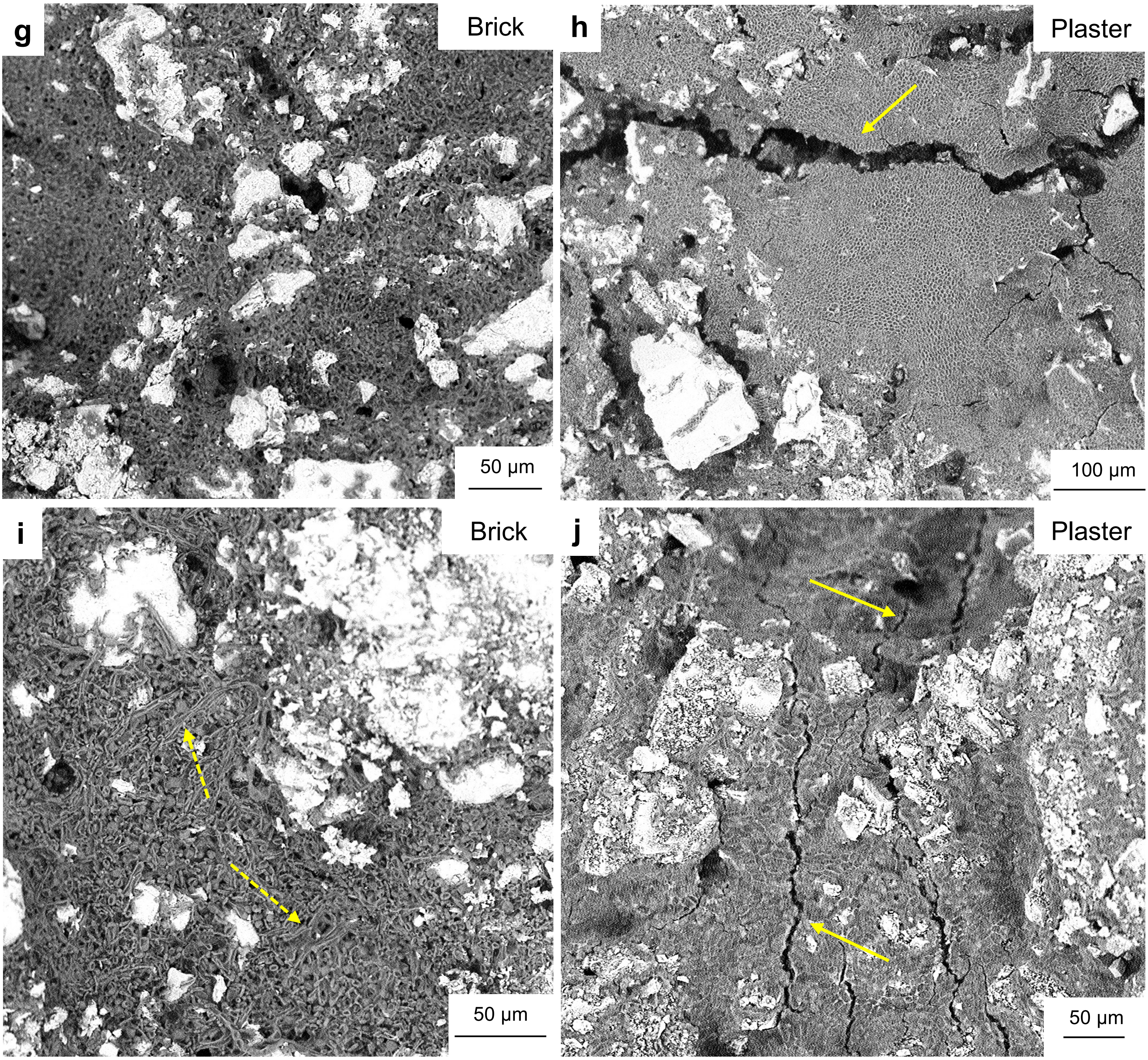

Figure S16 cont. Algal biofilms in SEM on brick and plaster substrates; g-h. *Diplosphaera chodatii* PNK021, i-j. *Stichococcus bacillaris* PNK040; solid arrows point at biofilm cracks, while dashed arrows point at directional development of *S. bacillaris* cells.
